# Supplementary material for: Shank3 modulates sleep and expression of circadian transcription factors
Source: eLife. 2019 Apr 11;8:e42819. doi: 10.7554/eLife.42819 (PMC6488297; doi:10.7554/eLife.42819)
Supplement: Source code 1. [file elife-42819-code1.pdf]

Shank3 Modulates Sleep and Expression of Circadian Transcription Factors differential expression

February 27, 2019

Contents

Description 1
- Importing data 2
- Plot PCA of log unnormalized data 2
- Control Genes 3
  - Negative control genes 3
  - positive control genes 4
- Normalizations 4
  - TMM Normalization 4
  - TMM + RUVs Normalization 6
- edgeR Differential Expression Analysis 8
  - Shank3 Home Cage control VS Wild Type Home Cage Controls 9
    - volcano plot 9
  - Shank3 Sleed Deprivation VS Wild Type Sleep Deprivation 11
    - volcano plot 11
  - DE TABLE + Positive Controls table 13
  - WTSD5 - WTHC5 positive controls 13
- Venn Diagram 14
  - KOHC5-WTHC5 vs KOSD5-WTSD5 14
- Heatmaps 15
  - Heatmap gene by bene 16
  - other heatmaps 19
- Group gene profiles 23
  - Group gene profiles by genotype 23
  - Group gene profiles by condition 24

Circadian Analysis LD-DD 25
- Analysis for activity 25
- Analysis for alpha 27
- Analysis for period 29

Circadian Analysis LD-LD 30
- Analysis for activity 30
- Analysis for alpha 32
- Analysis for period 34

Analysis for Spectral Data 35

Session Info 39

Description

This is the report of the analysis made for the paper *Shank3 Modulates Sleep and Expression of Circadian Transcription Factors* by *Ashley M. Ingiosi, Hannah Schoch, Taylor Wintler, Kristan G. Singletary, Dario Righelli, Leandro G. Roser, Elizabeth Medina, Davide Risso, Marcos G. Frank and Lucia Peixoto.*

Autism Spectrum Disorder (ASD) is the most prevalent neurodevelopmental disorder in the US that often co-presents with sleep problems. Sleep impairments in ASD predict the severity of ASD core diagnostic symptoms and have a considerable impact on the quality of life of caregivers. However, little is known about the underlying molecular mechanism(s) of sleep impairments in ASD. In this study we investigated the role of Shank3, a high confidence ASD gene candidate, in the regulation of sleep. We show that Shank3 mutant mice have problems falling asleep despite accumulating sleep pressure. Using RNA-seq we show that sleep deprivation doubles the differences in gene expression between mutants and wild types and downregulates circadian transcription factors Per3, Dec2, and Rev-erbα. Shank3 mutants also have trouble regulating locomotor activity in the absence of light. Overall, our study shows that Shank3 is an important modulator of sleep and circadian activity. # Differential Expression Analysis

Importing data

Importing data and filtering out those genes with cpm lesser than 1. We use the *filtered.data* method in *NOISeq* package.

```
countMatrix <- ReadDataFrameFromTsv(file.name.path="./data/refSEQ_countMatrix.txt")
```

```
## ./data/refSEQ_countMatrix.txt read from disk!
```

```
# head(countMatrix)

designMatrix <- ReadDataFrameFromTsv(file.name.path="./design/all_samples_short_names.tsv")
```

```
## ./design/all_samples_short_names.tsv read from disk!
```

```
# head(designMatrix)

filteredCountsProp <- filterLowCounts(counts.dataframe=countMatrix,
                                      is.normalized=FALSE,
                                      design.dataframe=designMatrix,
                                      cond.col.name="gcondition",
                                      method.type="Proportion")
```

```
## features dimensions before normalization: 27179
```

```
## Filtering out low count features...
## 14454 features are to be kept for differential expression analysis with filtering method 3
```

Plot PCA of log unnormalized data

PCA Plot of filtered not-normalized data.

```
PlotPCAPlotlyFunction(counts.data.frame=log1p(filteredCountsProp),
    design.matrix=designMatrix,
    shapeColname="condition", colorColname="genotype", xPCA="PC1", yPCA="PC2",
    plotly.flag=FALSE, show.plot.flag=TRUE, prefix.plot="Prop-Un-Norm")
```

```
## [1] FALSE
```

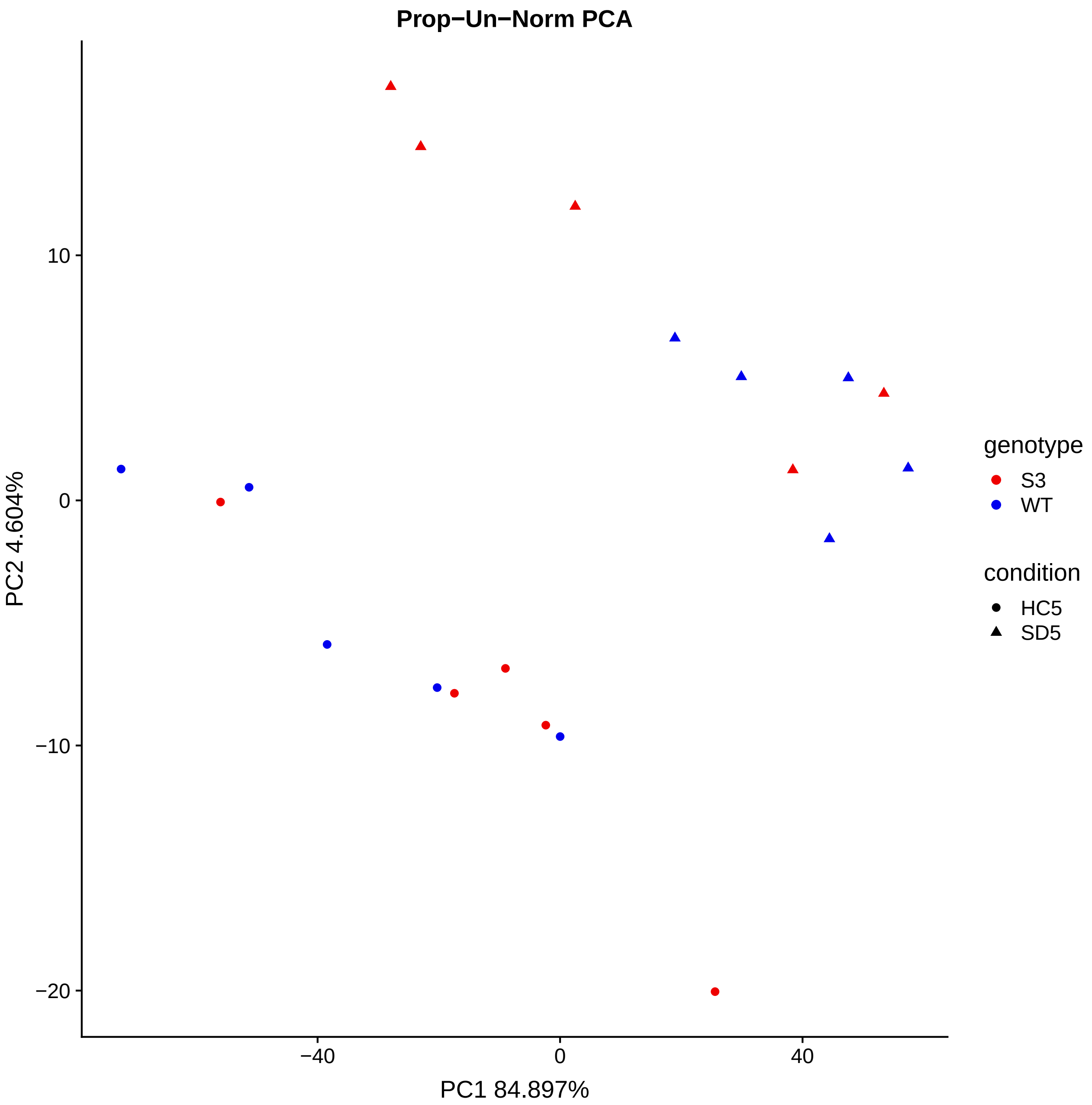

Control Genes

Negative control genes

Loading Negative Control Genes to normalize data

```
library(readxl)

sd.ctrls <- read_excel(path="./data/controls/Additional File 4 full list of BMC genomics SD&RS2.xlsx", s
sd.ctrls <- sd.ctrls[order(sd.ctrls$adj.P.Val),]

sd.neg.ctrls <- sd.ctrls[sd.ctrls$adj.P.Val > 0.9, ]

sd.neg.ctrls <- sd.neg.ctrls$`MGI Symbol`
```

```r
sd.neg.ctrls <- sd.neg.ctrls[-which(is.na(sd.neg.ctrls))]

int.neg.ctrls <- sd.neg.ctrls
int.neg.ctrls <- unique(int.neg.ctrls)
neg.map <- convertGenesViaMouseDb(gene.list=int.neg.ctrls, fromType="SYMBOL",
                                "ENTREZID")
# sum(is.na(neg.map$ENTREZID))
neg.ctrls.entrez <- as.character(neg.map$ENTREZID)


ind.ctrls <- which(rownames(filteredCountsProp) %in% neg.ctrls.entrez)
counts.neg.ctrls <- filteredCountsProp[ind.ctrls,]
```

positive control genes

Loading Positive Control Genes to detect them during the differential expression step.

```r
## sleep deprivation
sd.lit.pos.ctrls <- read_excel("./data/controls/SD_RS_PosControls_final.xlsx",
                        sheet=1)
colnames(sd.lit.pos.ctrls) <- sd.lit.pos.ctrls[1,]
sd.lit.pos.ctrls <- sd.lit.pos.ctrls[-1,]


sd.est.pos.ctrls <- read_excel("./data/controls/SD_RS_PosControls_final.xlsx",
                        sheet=3)

sd.pos.ctrls <- cbind(sd.est.pos.ctrls$`MGI Symbol`, "est")
sd.pos.ctrls <- rbind(sd.pos.ctrls, cbind(sd.lit.pos.ctrls$Gene, "lit"))

sd.pos.ctrls <- sd.pos.ctrls[-which(duplicated(sd.pos.ctrls[,1])),]
sd.pos.ctrls <- sd.pos.ctrls[-which(is.na(sd.pos.ctrls[,1])),]
```

Normalizations

TMM Normalization

Normalizing data with *TMM*, as implemented in *edgeR* package, and plotting a PCA and an RLE plot of them.

```r
normPropCountsUqua <- NormalizeData(data.to.normalize=filteredCountsProp,
                                    norm.type="tmm",
                                    design.matrix=designMatrix)

PlotPCAPlotlyFunction(counts.data.frame=log1p(normPropCountsUqua),
                    design.matrix=designMatrix, shapeColname="condition",
                    colorColname="genotype", xPCA="PC1", yPCA="PC2",
                    plotly.flag=FALSE, show.plot.flag=TRUE,
                    prefix.plot="TMM-Norm")
```

```
## [1] FALSE
```

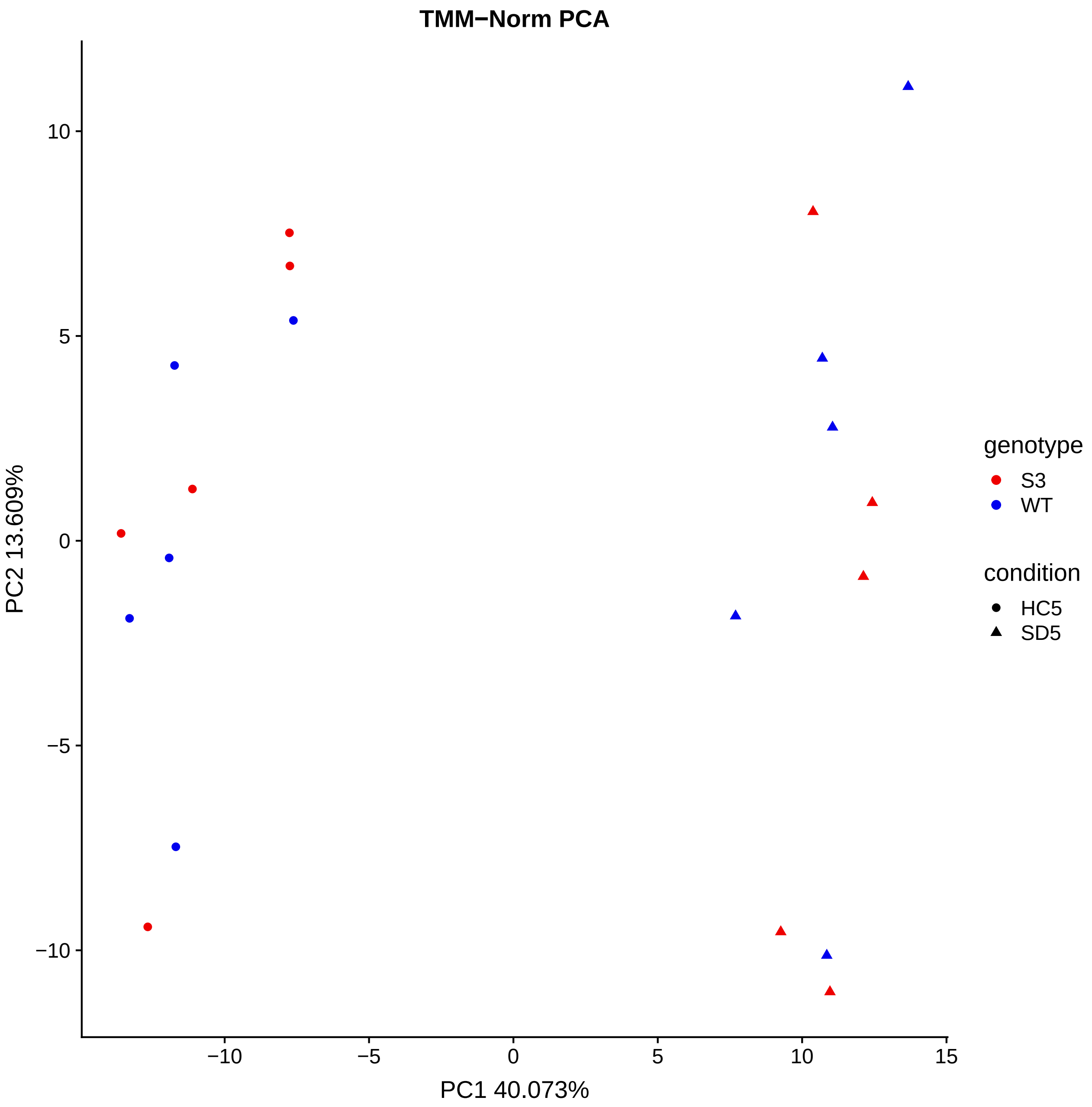


```
pal <- RColorBrewer::brewer.pal(9, "Set1")
plotRLE(as.matrix(normPropCountsUqua), outline=FALSE, col=pal[designMatrix$gcondition])
```

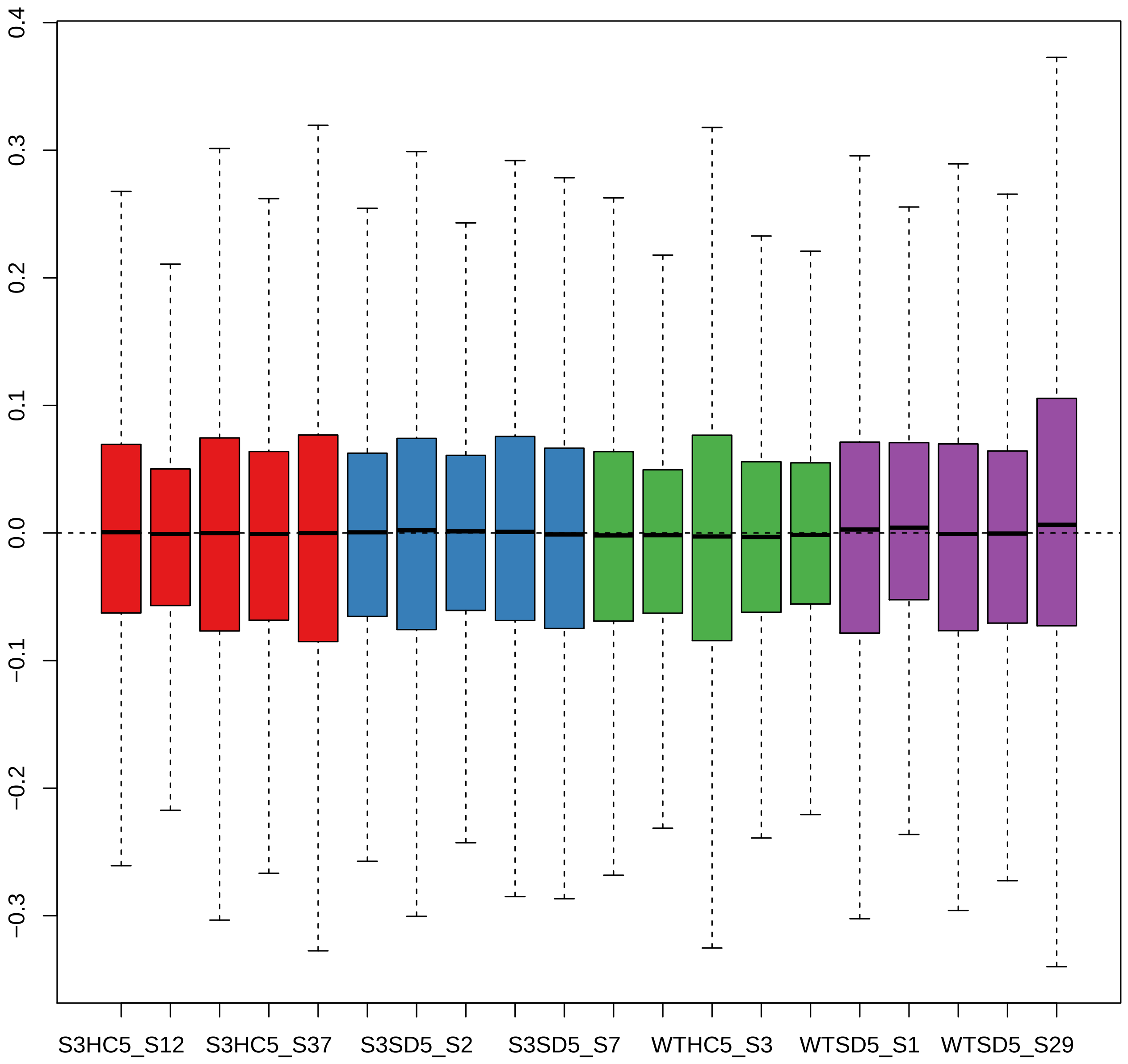

TMM + RUVs Normalization

Applying a *RUVs* method of *RUVSeq* package on normalized data, in order to adjust the counts for the unwanted variation. And of corse we plot a PCA and an RLE plot on these data.

```
library(RUVSeq)
neg.ctrl.list <- rownames(counts.neg.ctrls)
groups <- makeGroups(designMatrix$gcondition)
ruvedSExprData <- RUVs(as.matrix(round(normPropCountsUqua)), cIdx=neg.ctrl.list,
                       scIdx=groups, k=5)

normExprData <- ruvedSExprData$normalizedCounts

ggp <- PlotPCAPlotlyFunction(counts.data.frame=log1p(normExprData),
                     design.matrix=designMatrix, shapeColname="condition",
                     colorColname="genotype", xPCA="PC1", yPCA="PC2",
                     plotly.flag=FALSE, show.plot.flag=FALSE, save.plot=FALSE,
```

```
                    prefix.plot=NULL)
```

```
## [1] FALSE
```

```
print(ggp)
```

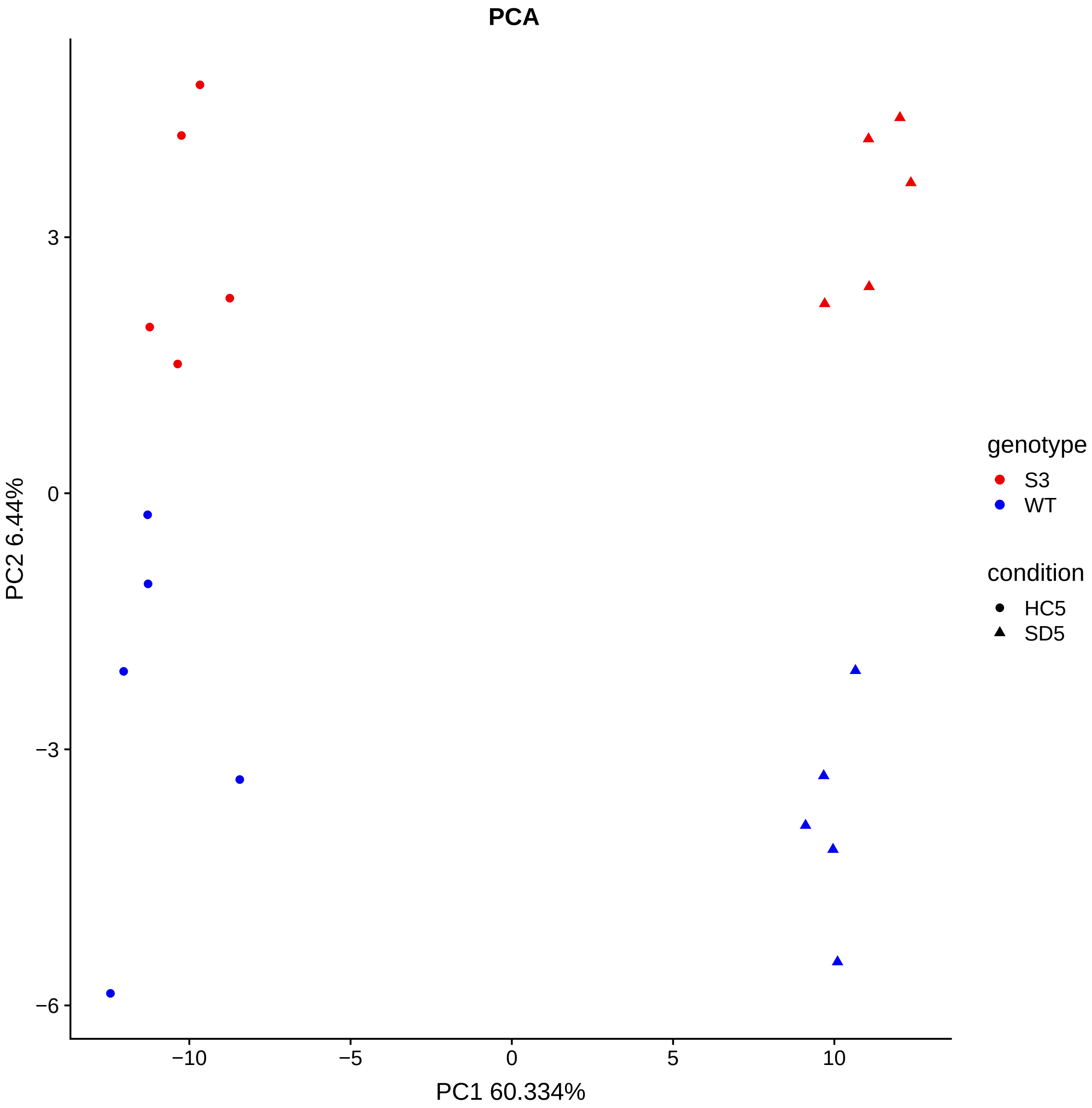


```
dir.create("plots")
save_plot(filename="plots/PCA.pdf", plot=ggp)

pal <- RColorBrewer::brewer.pal(9, "Set1")
plotRLE(normExprData, outline=FALSE, col=pal[designMatrix$gcondition])
```

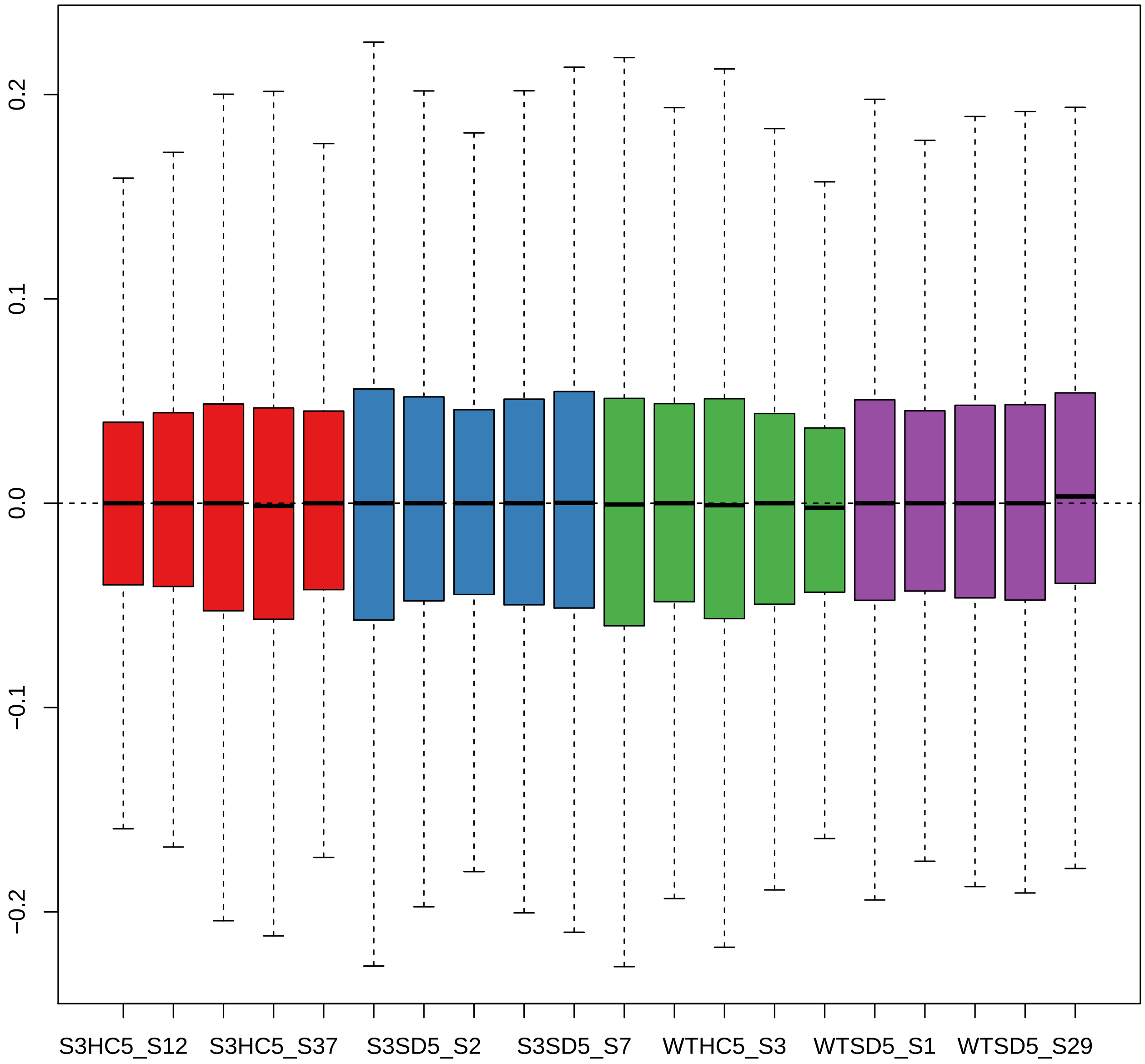

edgeR Differential Expression Analysis

Making differential expression analysis with *edgeR* package on four different contrasts.

Here is a brief legend:

- WTHC5: Wild Type Home Cage Control 5 days
- WTSD5: Wild Type Sleep Deprivation 5 days.
- KOHC5: Knock Out Home Cage Control 5 days.
- KOSD5: Knock Out Sleep Deprivation 5 days.

```
padj.thr <- 0.05
venn.padgj.thr <- 0.1
desMat <- cbind(designMatrix, ruvedSExprData$W)
colnames(desMat) <- c(colnames(designMatrix), colnames(ruvedSExprData$W))

cc <- c("S3HC5 - WTHC5", "S3SD5 - WTSD5", "WTSD5 - WTHC5")
```

```r
rescList1 <- applyEdgeR(counts=filteredCountsProp, design.matrix=desMat,
                        factors.column="gcondition",
                        weight.columns=c("W_1", "W_2", "W_3", "W_4", "W_5"),
                        contrasts=cc, useIntercept=FALSE, p.threshold=1,
                        is.normalized=FALSE, verbose=TRUE)
names <- names(rescList1)
rescList1 <- lapply(seq_along(rescList1), function(i)
{
    attachMeans(normalized.counts=normExprData, design.matrix=desMat,
                factor.column="gcondition", contrast.name=names(rescList1)[i],
                de.results=rescList1[[i]])
})
names(rescList1) <- names
```

Shank3 Home Cage control VS Wild Type Home Cage Controls

volcano plot

A volcano plot of differential expressed genes.

```r
res.o.map1 <- convertGenesViaMouseDb(gene.list=rownames(rescList1[[1]]),
                            fromType="ENTREZID")

res.o <- attachGeneColumnToDf(mainDf=rescList1[[1]],
                                genesMap=res.o.map1,
                                rowNamesIdentifier="ENTREZID",
                                mapFromIdentifier="ENTREZID",
                                mapToIdentifier="SYMBOL")
WriteDataFrameAsTsv(data.frame.to.save=res.o,
                    file.name.path=paste0(names(rescList1)[1], "_edgeR"))

vp <- luciaVolcanoPlot(res.o, prefix=names(rescList1)[1],
                        positive.controls.df=NULL,
                        threshold=padj.thr, plotly.flag=FALSE)
print(vp)
```

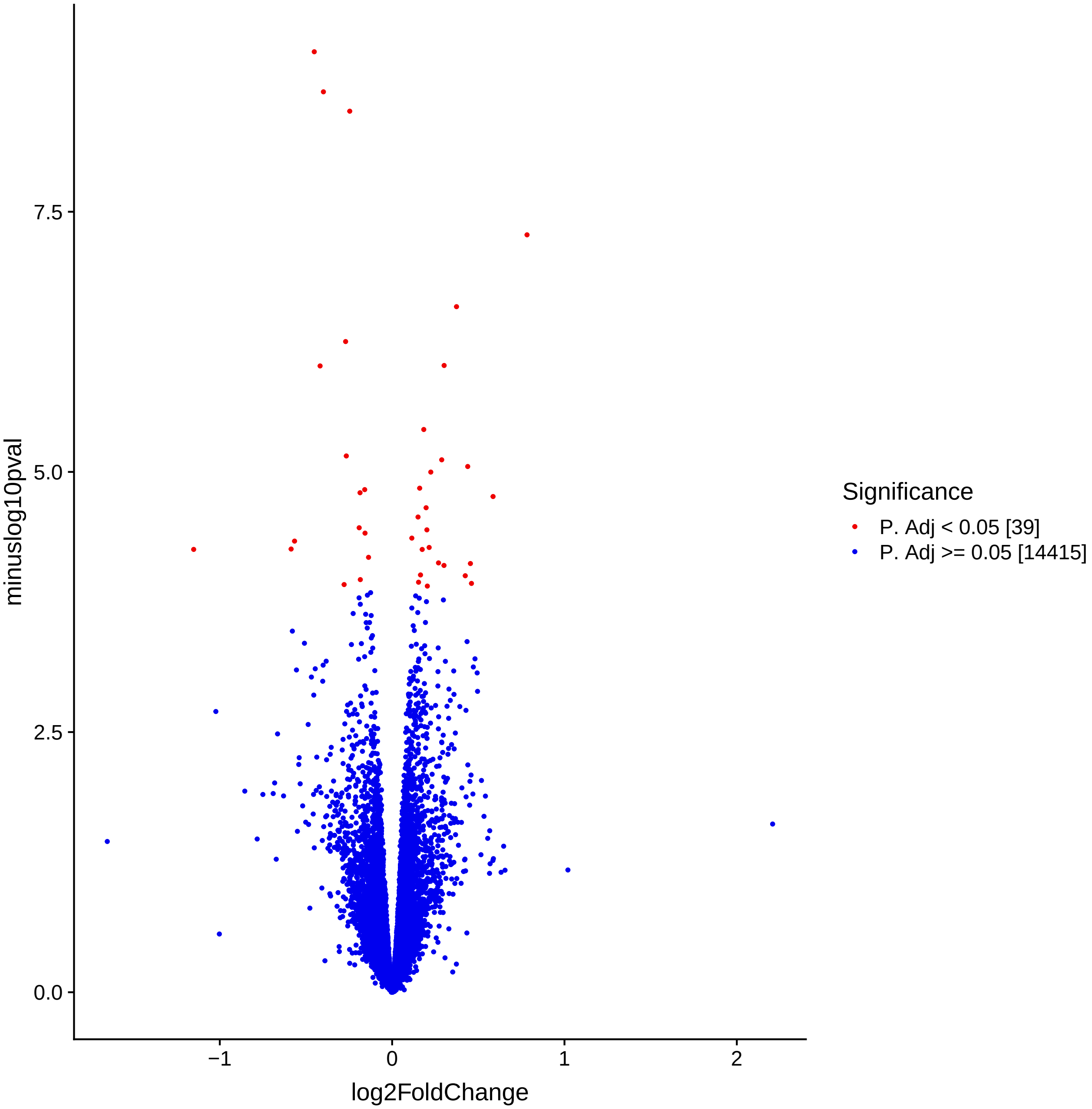


```
de <- sum(res.o$FDR < padj.thr)
nde <- sum(res.o$FDR >= padj.thr)
detable <- cbind(de,nde)
rownames(detable) <- names(rescList1)[1]
ddetable <- detable

tot.ctrls <- dim(sd.pos.ctrls)[1]
idx.pc <- which(tolower(res.o$gene) %in% tolower(sd.pos.ctrls[,1]))
tot.pc.de <- sum(res.o$FDR[idx.pc] < padj.thr)
tot.pc.nde <- length(idx.pc) - tot.pc.de

wt <- res.o[which(res.o$FDR < padj.thr),]
wt.sign.genes.entrez <- rownames(res.o)[which(res.o$FDR < venn.padgj.thr)]
```

```r
kowthc5 <- res.o[which(res.o$FDR < padj.thr),]
kowthc5.sign.genes.entrez <- rownames(res.o)[which(res.o$FDR < venn.padgj.thr)]
```

Shank3 Sleed Deprivation VS Wild Type Sleep Deprivation

volcano plot

A volcano plot of differential expressed genes.

```r
rs2.o.map <- convertGenesViaMouseDb(gene.list=rownames(rescList1[[2]]),
                            fromType="ENTREZID")

res.rs2.o <- attachGeneColumnToDf(mainDf=rescList1[[2]],
                                genesMap=rs2.o.map,
                                rowNamesIdentifier="ENTREZID",
                                mapFromIdentifier="ENTREZID",
                                mapToIdentifier="SYMBOL")
WriteDataFrameAsTsv(data.frame.to.save=res.rs2.o,
                    file.name.path=paste0(names(rescList1)[2], "_edgeR"))

vp <- luciaVolcanoPlot(res.rs2.o, positive.controls.df=NULL,
                       prefix=names(rescList1)[2],
                       threshold=padj.thr, plotly.flag=FALSE)
print(vp)
```

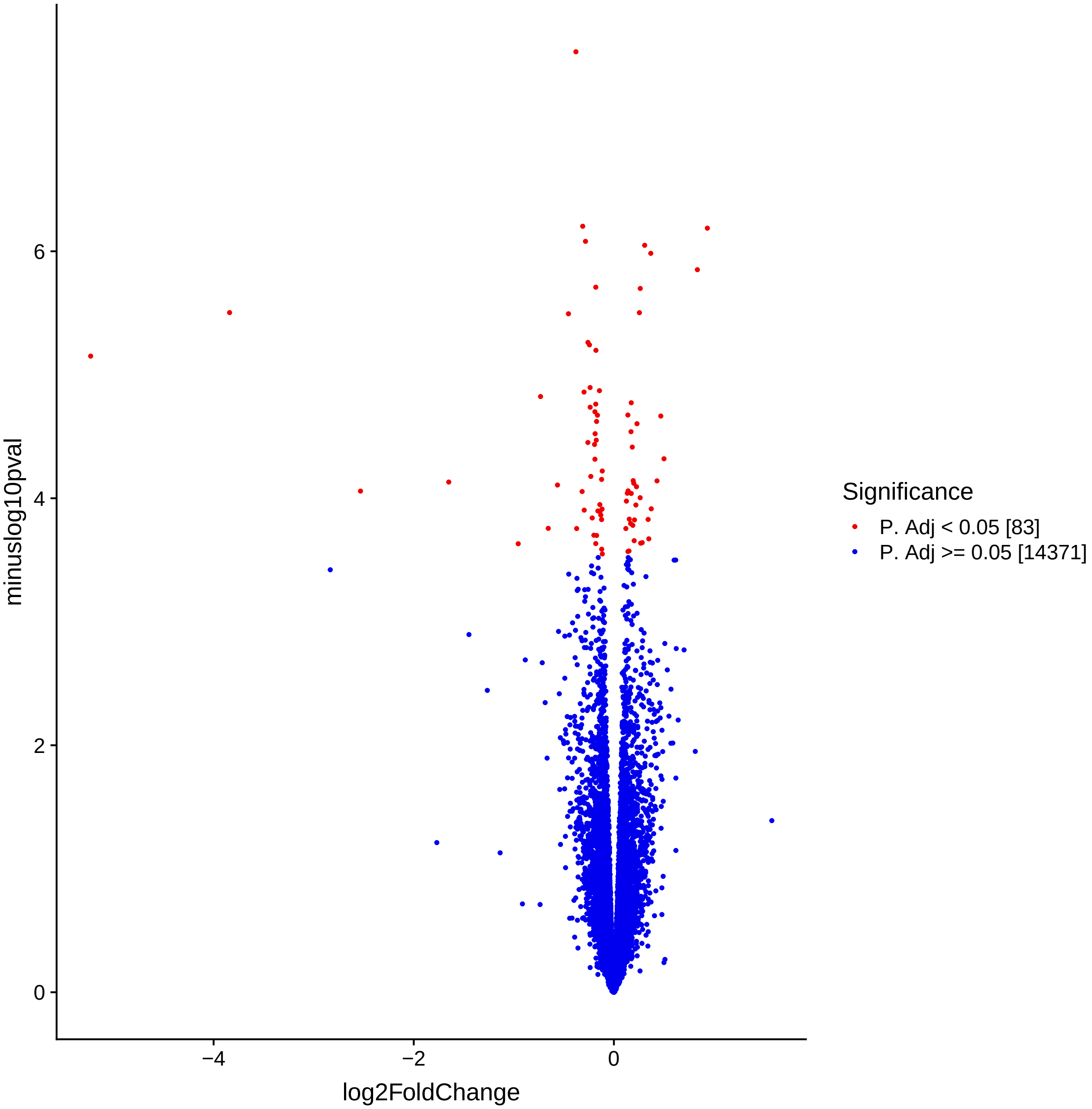


```
de <- sum(res.rs2.o$FDR < padj.thr)
nde <- sum(res.rs2.o$FDR >= padj.thr)
detable <- cbind(de,nde)
rownames(detable) <- names(rescList1)[2]
ddetable <- rbind(ddetable, detable)
pos.df <- cbind(tot.ctrls, tot.pc.de, tot.pc.nde)
colnames(pos.df) <- c("total_p.ctrl", "p.ctrl_de_mapped",
                    "p.ctrl_notde_mapped")
rownames(pos.df) <- names(rescList1)[2]

kowtsd5 <- res.rs2.o[which(res.rs2.o$FDR < padj.thr),]
kowtsd5.sign.genes.entrez <- rownames(res.rs2.o)[which(res.rs2.o$FDR < venn.padgj.thr)]
```

DE TABLE + Positive Controls table

We present a summarization of the results. The first table is a summarization on how many genes are Differentially Expressed. The second table explains on the first column how many positive controls we have, on the second column how many positive controls have been identified over the differentially expressed genes, and, finally, on the third column how many positive controls have beed identified on the NOT differentially expressed genes.

```
ddetable
```

```
##                de   nde
## S3HC5 - WTHC5 39 14415
## S3SD5 - WTSD5 83 14371
```

```
pos.df
```

```
##                total_p.ctrl p.ctrl_de_mapped p.ctrl_notde_mapped
## S3SD5 - WTSD5           579                3                 548
```

WTSD5 - WTHC5 positive controls

```
res.o.map.wtsd <- convertGenesViaMouseDb(gene.list=rownames(rescList1[["WTSD5 - WTHC5"]]),
                            fromType="ENTREZID")

res.o.wt.sd <- attachGeneColumnToDf(mainDf=rescList1[["WTSD5 - WTHC5"]],
                                genesMap=res.o.map.wtsd,
                                rowNamesIdentifier="ENTREZID",
                                mapFromIdentifier="ENTREZID",
                                mapToIdentifier="SYMBOL")


WriteDataFrameAsTsv(data.frame.to.save=res.o,
                    file.name.path=paste0(names(rescList1)[3], "_edgeR"))

vp <- luciaVolcanoPlot1(res.o.wt.sd, sd.pos.ctrls, prefix=names(rescList1)[3],
                    threshold=padj.thr, plotly.flag=FALSE)
print(vp)
```

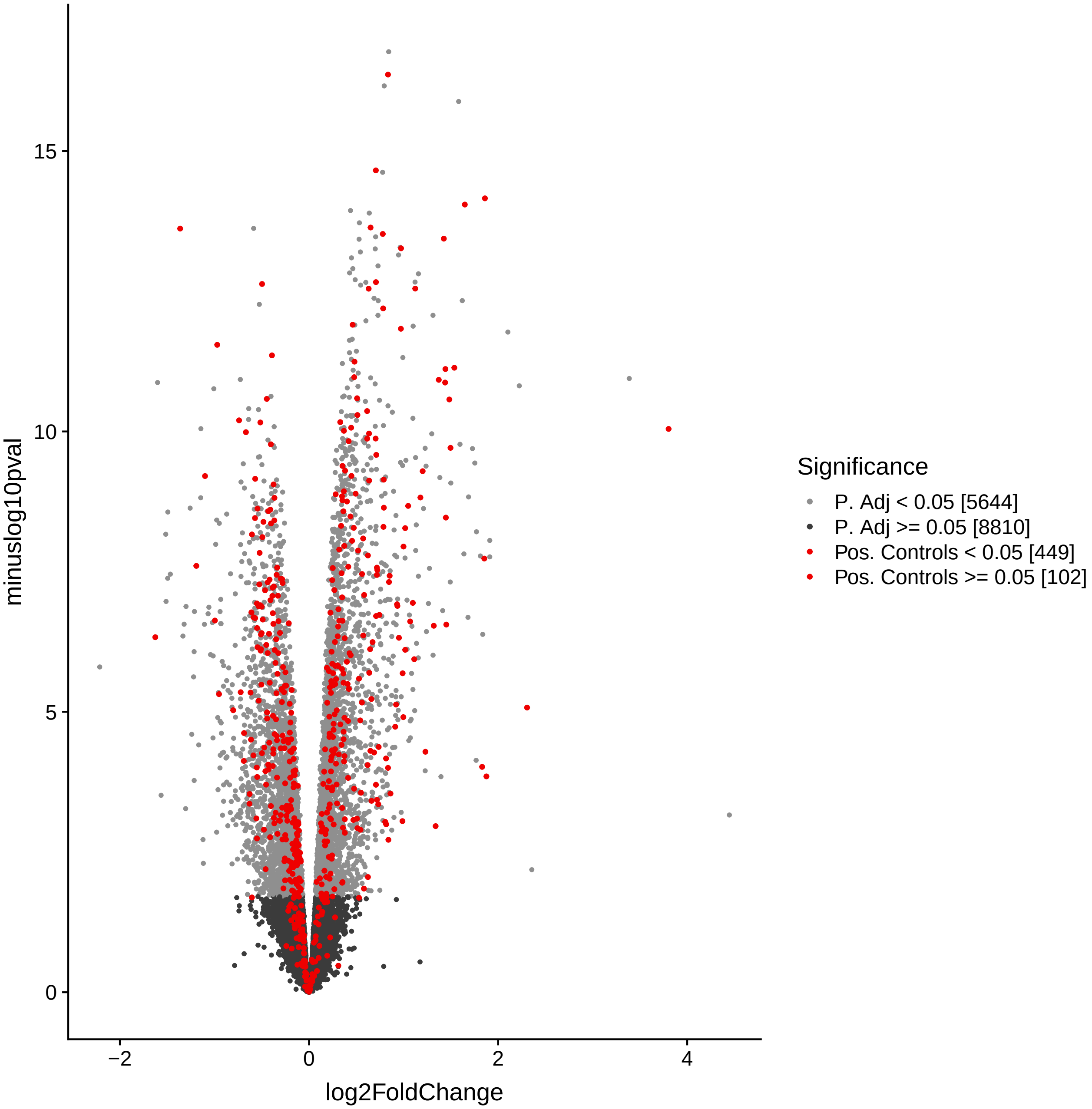

Venn Diagram

KOHC5-WTHC5 vs KOSD5-WTSD5

We take the results of the two contrasts. *Knock Out Sleed Deprivation VS Wild Type Sleep Deprivation* and *Knock Out Home Cage control VS Wild Type Home Cage Controls* . And plot the results in a Venn Diagram

```
source("./R/venn2.R")

gene.map <- convertGenesViaMouseDb(gene.list=rownames(normExprData),
                                    fromType="ENTREZID", toType="SYMBOL")
venn <- Venn2de(x=kowthc5.sign.genes.entrez, y=kowtsd5.sign.genes.entrez,
        label1="S3HC5_WTHC5", label2="S3SD5_WTSD5",
        title="S3HC5_WTHC5 venn S3SD5_WTSD5", plot.dir="./",
```

```
        conversion.map=gene.map)
```

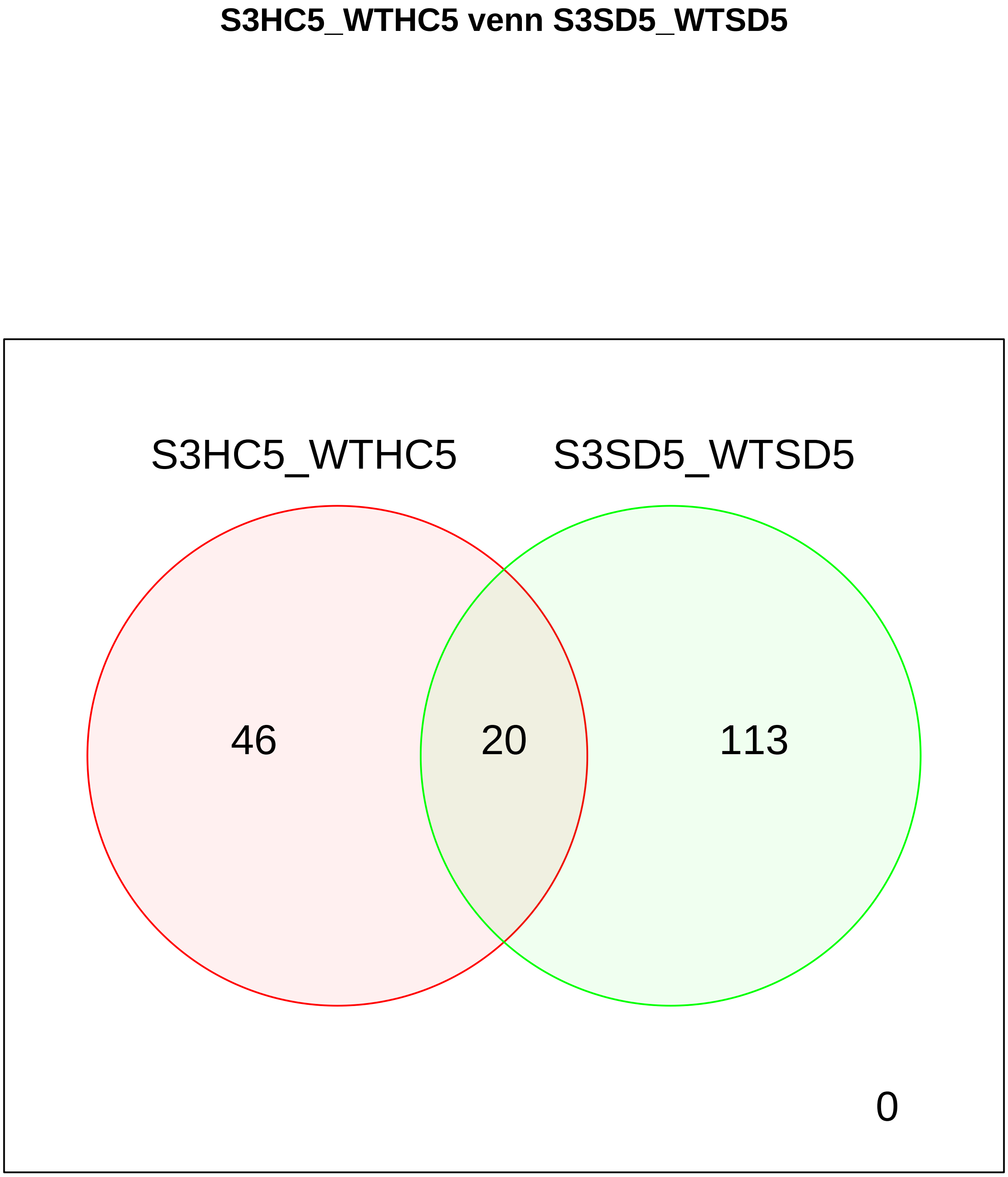

Heatmaps

Setting up the data structures for the heatmps.

```
source("./R/heatmapFunctions.R")
de.genes.entr <- union(rownames(venn$int), rownames(venn$XnoY))
de.genes.entr <- union(de.genes.entr, rownames(venn$YnoX))

gene.map <- convertGenesViaMouseDb(gene.list=de.genes.entr,
```

```
                              fromType="ENTREZID")
de.genes.symb <- attachGeneColumnToDf(as.data.frame(de.genes.entr,
                                                    row.names=de.genes.entr),
                                      genesMap=gene.map,
                                      rowNamesIdentifier="ENTREZID",
                                      mapFromIdentifier="ENTREZID",
                                      mapToIdentifier="SYMBOL")

# de.genes.symb[which(is.na(de.genes.symb$gene)),]
de.genes.symb$gene[which(de.genes.symb$de.genes.entr=="100039826")]  <- "Gm2444" ## not annotated in nc
de.genes.symb$gene[which(de.genes.symb$de.genes.entr=="210541")]  <- "Entrez:210541" ## not annotated i


de.genes.counts <- normExprData[match(de.genes.symb$de.genes.entr, rownames(normExprData)),]
rownames(de.genes.counts) <- de.genes.symb$gene

de.gene.means <- computeGeneMeansOverGroups(counts=de.genes.counts,
                              design=designMatrix, groupColumn="gcondition")

library(gplots)
library(clusterExperiment)
color.palette = clusterExperiment::seqPal3#c("black", "yellow")
pal <- colorRampPalette(color.palette)(n = 1000)


library(pheatmap)
filter2 <- rowMeans(de.gene.means)>0
filter <- apply(de.gene.means, 1, function(x) log(x[4]/x[3]) * log(x[2]/x[1]) < 0)
filter[is.na(filter)] <- FALSE
```

Heatmap gene by bene

```
gene_names <- c("Nr1d1", "Fabp7", "Per3", "Jun", "Elk1", "Fosl2", "Mapk1",
                "Mapk3", "Mapk11", "Hmgcr", "Insig1", "Nfil3", "Stat4",
                "Kcnv1", "Kcnk1", "Kcnk2", "Dusp10", "Dusp3", "Ptprj",
                "Cntn1", "Ntrk2", "Reln", "Sema3a", "Tef", "Hlf", "Nr1d1",
                "Prkab2", "Bhlhe41", "Slc6a15", "Slc22a4", "Slc24a4")
idx <- which(!(rownames(de.genes.counts) %in% gene_names))
de.genes.counts1 <- de.genes.counts
rownames(de.genes.counts1)[idx] <- ""
ann.col <- desMat[, c(1:2)]
de.heatmap <- de.genes.counts[filter2,]
set.seed(0)
heatmap_data <- t(scale(t(log(de.heatmap+1)), center = TRUE, scale = FALSE))
ph1 <- pheatmap(heatmap_data, cluster_cols=TRUE, scale="none",
            color=pal, border_color=NA, fontsize_row=10, kmeans_k=3, annotation_col=ann.col)
```

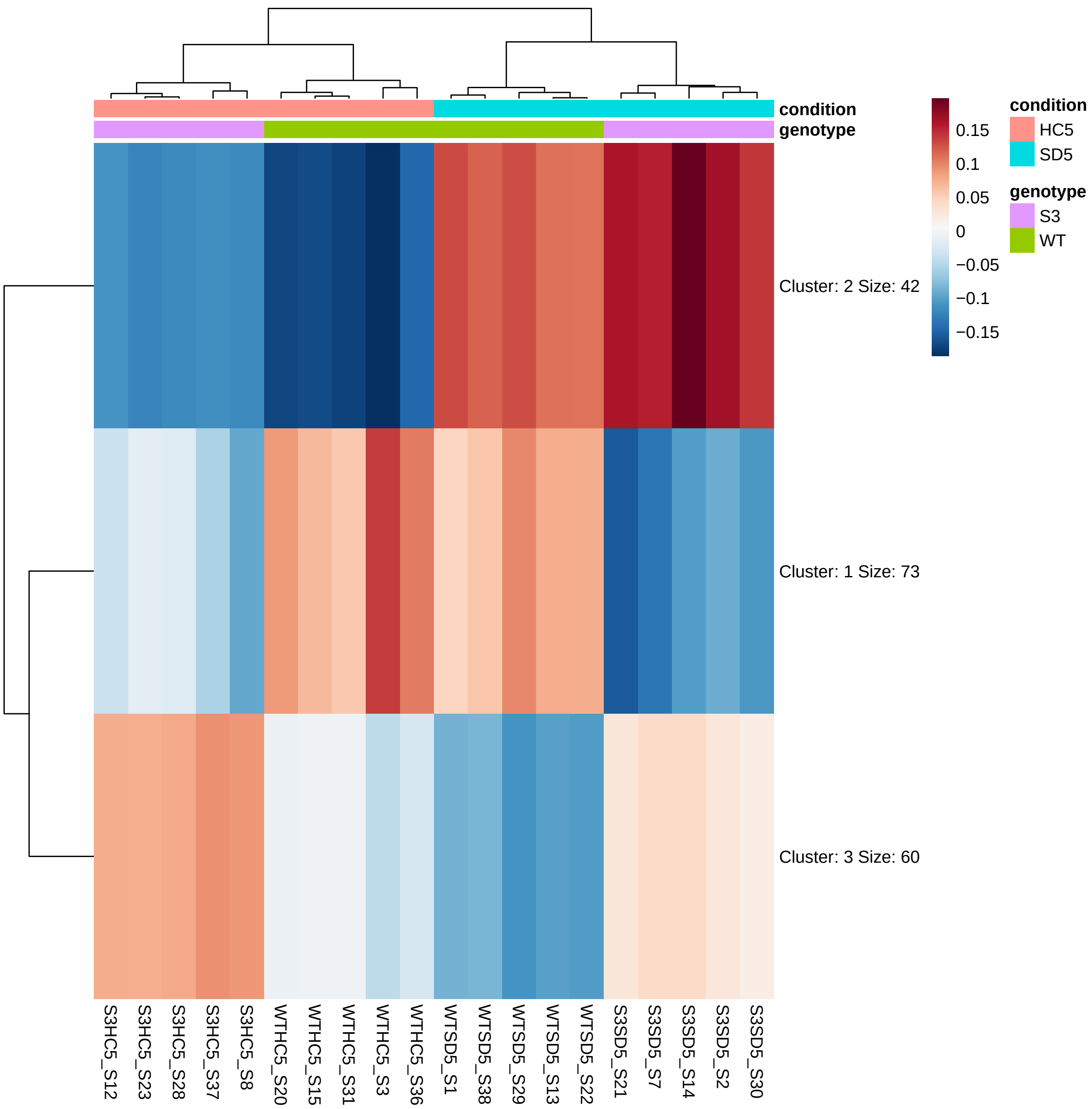


```
clusterized.genes <- as.data.frame(ph1$kmeans$cluster)

gene.map <- convertGenesViaMouseDb(gene.list=rownames(clusterized.genes), fromType="SYMBOL")
converted.clusterized.gens <- attachGeneColumnToDf(mainDf=clusterized.genes, genesMap=gene.map,
                    rowNamesIdentifier="SYMBOL", mapFromIdentifier="SYMBOL", mapToIdentifier="ENTREZID"

converted.clusterized.gens$gene[which(rownames(converted.clusterized.gens)=="Gm2444")]  <- "100039826"
converted.clusterized.gens$gene[which(rownames(converted.clusterized.gens)=="Entrez:210541")]  <- "2105
converted.clusterized.gens <- converted.clusterized.gens[order(converted.clusterized.gens$`ph1$kmeans$c

save_pheatmap_pdf(filename="plots/heatmap_kmeans_k3.pdf", plot=ph1, width=20, height=20)
```

```
## pdf
##   2
```

```r
WriteDataFrameAsTsv(data.frame.to.save=converted.clusterized.gens, file.name.path="plots/clustered_gene

ord.de.genes.counts <- de.heatmap[match(rownames(converted.clusterized.gens), rownames(de.heatmap)),]
idx <- which(!(rownames(ord.de.genes.counts) %in% gene_names))

rownames(ord.de.genes.counts)[idx] <- ""
gaps.row <- c()
for(i in c(1:3))
{
    li <- length(which(converted.clusterized.gens$`ph1$kmeans$cluster`==i))
    l <- ifelse(i!=1, gaps.row[i-1]+li, li)
    gaps.row <- c(gaps.row, l)
}

heatmap_data_scaled <- t(scale(t(log(ord.de.genes.counts+1)), center = TRUE, scale = TRUE))

library(dendextend)
column_dend <- as.dendrogram(hclust(dist(t(heatmap_data_scaled))))
ord <- labels(column_dend)
ord[11:15] <- labels(column_dend)[16:20]
ord[16:20] <- labels(column_dend)[11:15]
column_dend <- rotate(column_dend, ord)

ph1 <- pheatmap(heatmap_data_scaled, cluster_cols=as.hclust(column_dend), scale="none",
            color=pal, border_color=NA, fontsize_row=9, fontsize_col=9, cluster_rows=FALSE,
            annotation_col=ann.col, gaps_row=gaps.row)
```

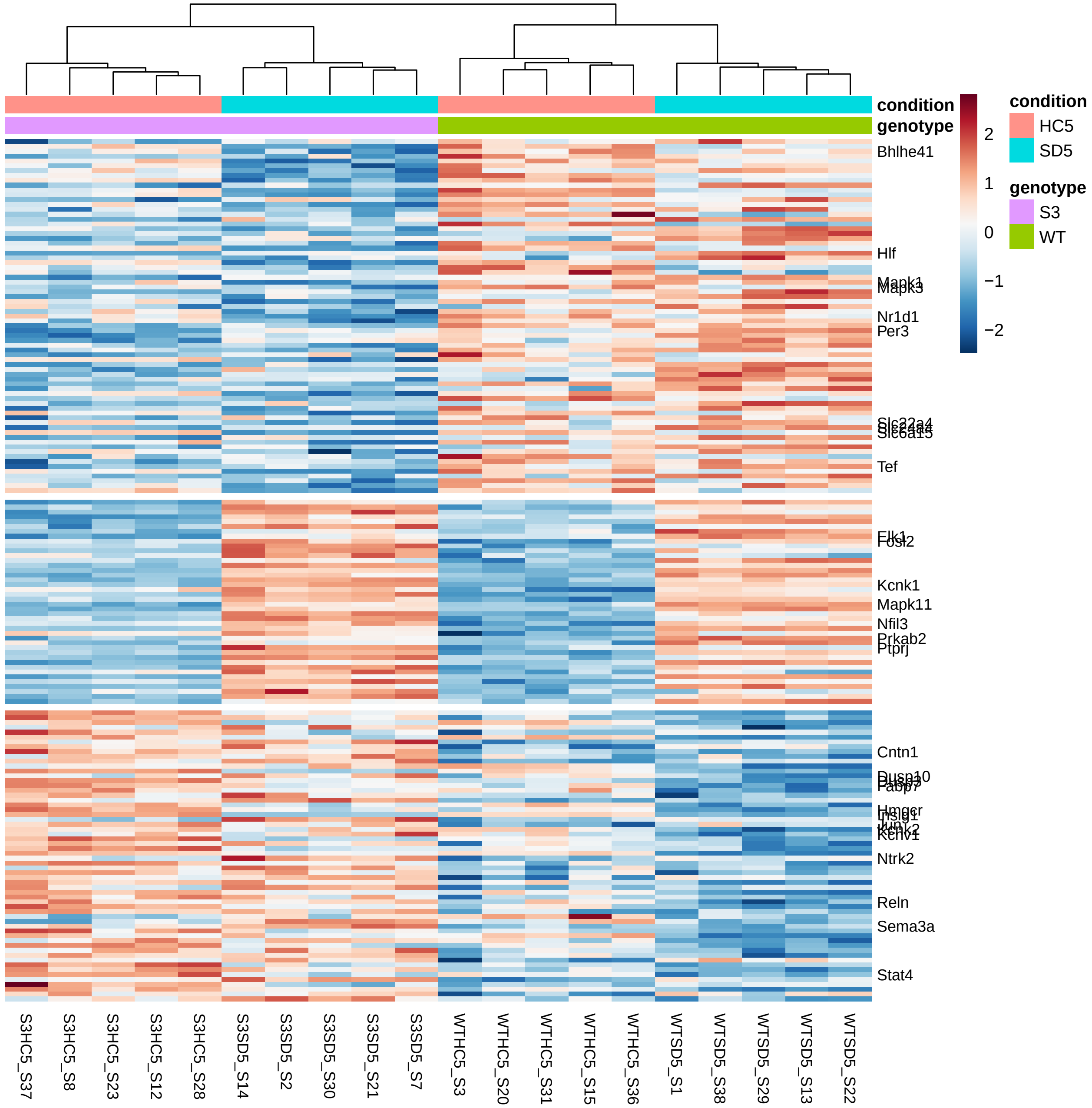


```r
save_pheatmap_pdf(filename="plots/heatmap_gg_k3.pdf", plot=ph1, width=20, height=20)
```

```
## pdf
##   2
```

other heatmaps

```r
heatmap_data <- t(scale(t(log(ord.de.genes.counts+1)), center = TRUE, scale = FALSE))

ph1 <- pheatmap(heatmap_data, cluster_cols=as.hclust(column_dend), scale="none",
          color=pal, border_color=NA, fontsize_row=9, fontsize_col=9, cluster_rows=FALSE,
          annotation_col=ann.col, gaps_row=gaps.row,
          breaks = c(min(heatmap_data), seq(quantile(as.vector(heatmap_data), .01), quantile(as.vecto
```

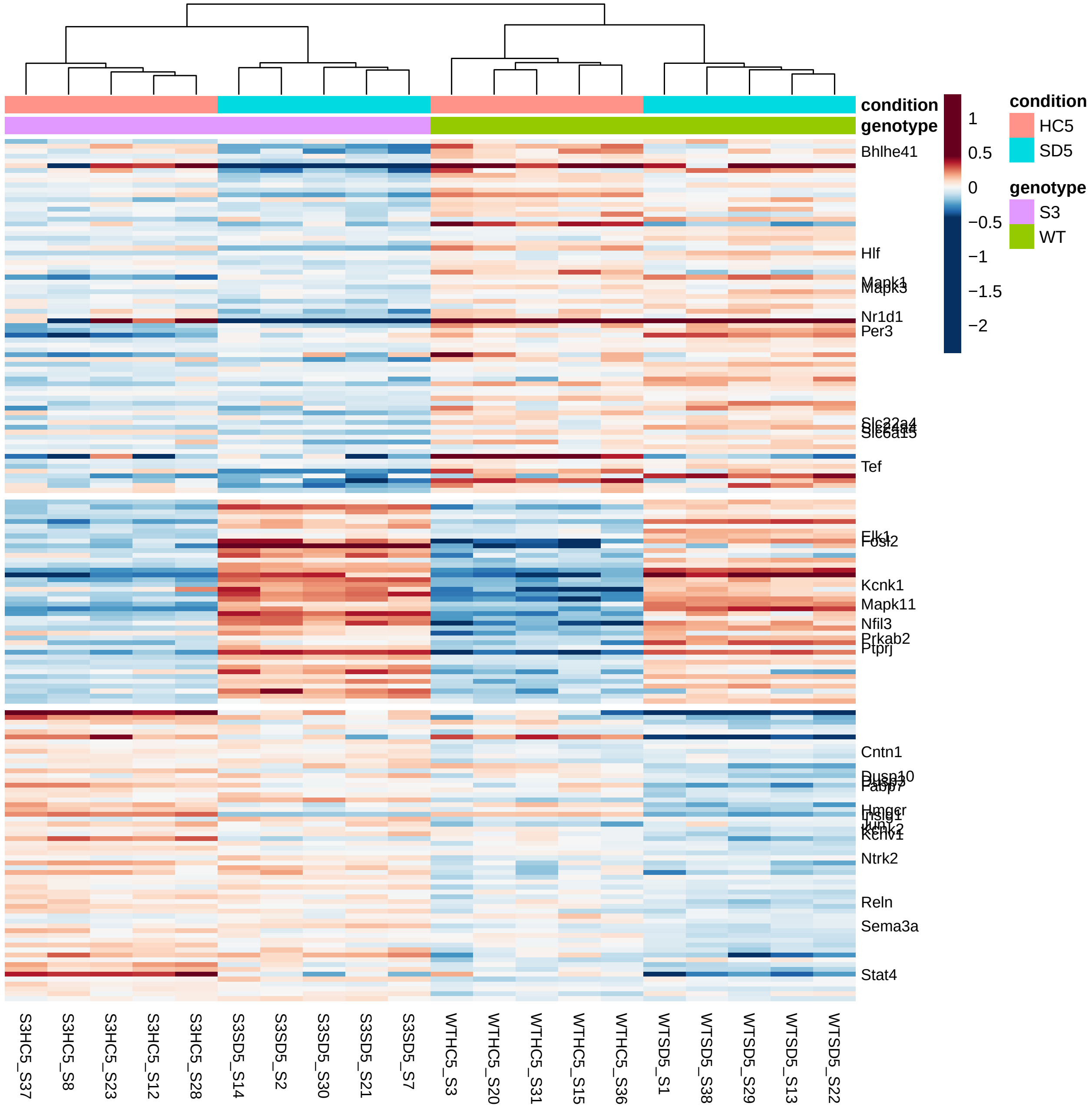


```r
save_pheatmap_pdf(filename="plots/heatmap_gg_k3_no_scale.pdf", plot=ph1, width=20, height=20)
```

```
## pdf
##   2
```

```r
## Only SD samples
heatmap_data <- t(scale(t(log(de.heatmap[, grep("^SD", desMat[,2])]+1)), center = TRUE, scale = FALSE))
ph1 <- pheatmap(heatmap_data, cluster_cols=TRUE, scale="none",
          color=pal, border_color=NA, fontsize_row=10, kmeans_k=2, annotation_col=ann.col)
```

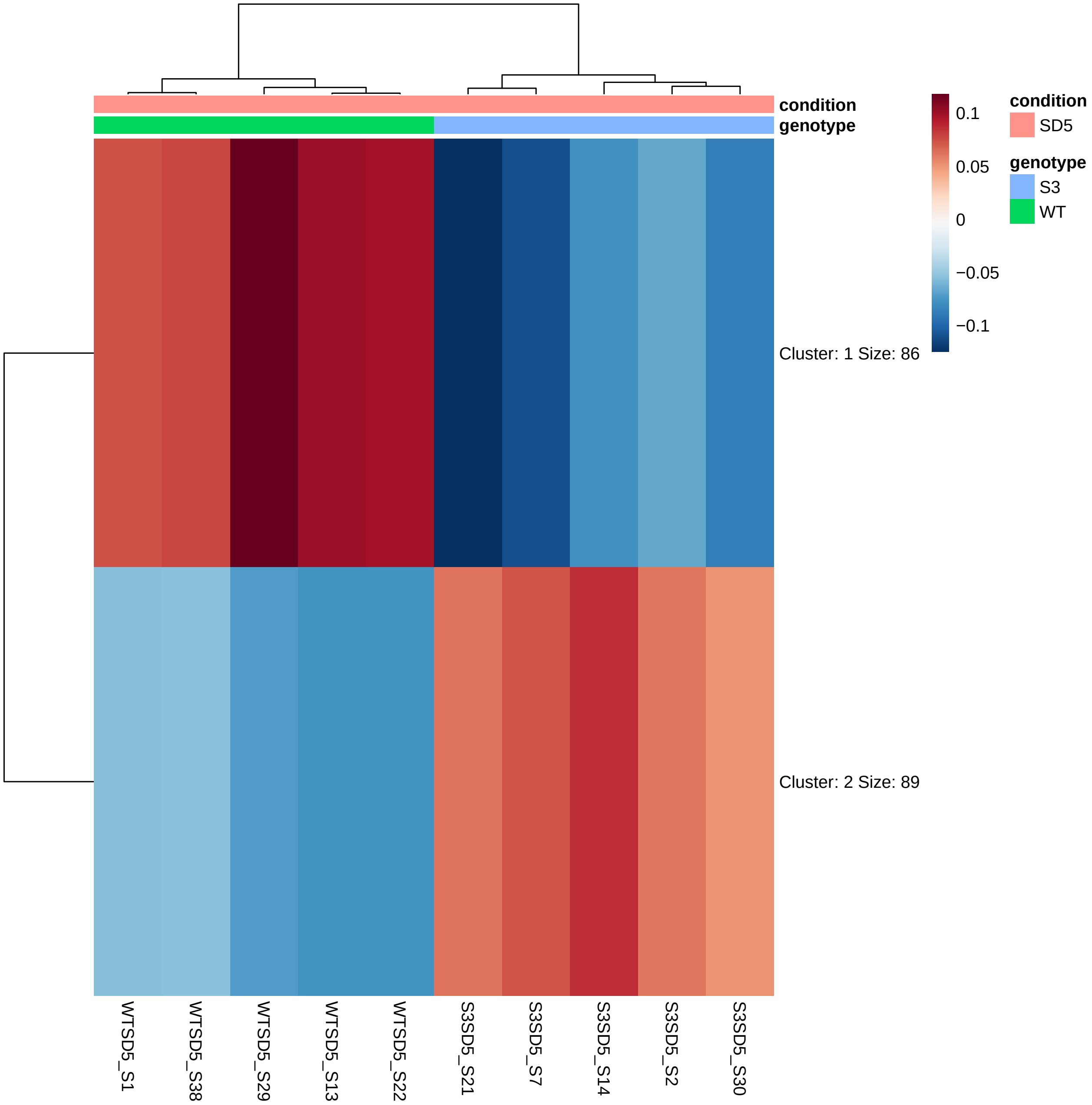


```
clusterized.genes <- as.data.frame(ph1$kmeans$cluster)

gene.map <- convertGenesViaMouseDb(gene.list=rownames(clusterized.genes), fromType="SYMBOL")
converted.clusterized.gens <- attachGeneColumnToDf(mainDf=clusterized.genes, genesMap=gene.map,
                    rowNamesIdentifier="SYMBOL", mapFromIdentifier="SYMBOL", mapToIdentifier="ENTREZID"

converted.clusterized.gens$gene[which(rownames(converted.clusterized.gens)=="Gm2444")]  <- "100039826"
converted.clusterized.gens$gene[which(rownames(converted.clusterized.gens)=="Entrez:210541")]  <- "2105
converted.clusterized.gens <- converted.clusterized.gens[order(converted.clusterized.gens$`ph1$kmeans$c

ord.de.genes.counts <- de.heatmap[match(rownames(converted.clusterized.gens), rownames(de.heatmap)),]
idx <- which(!(rownames(ord.de.genes.counts) %in% gene_names))
```

```
rownames(ord.de.genes.counts)[idx] <- ""
gaps.row <- c()
for(i in c(1:2))
{
    li <- length(which(converted.clusterized.gens$`ph1$kmeans$cluster`==i))
    l <- ifelse(i!=1, gaps.row[i-1]+li, li)
    gaps.row <- c(gaps.row, l)
}

heatmap_data <- t(scale(t(log(ord.de.genes.counts[, grep("^SD", desMat[,2])]+1)), center = TRUE, scale =

ph1 <- pheatmap(heatmap_data, cluster_cols=TRUE, scale="none",
            color=pal, border_color=NA, fontsize_row=9, fontsize_col=9, cluster_rows=FALSE,
            annotation_col=ann.col, gaps_row=gaps.row)
```

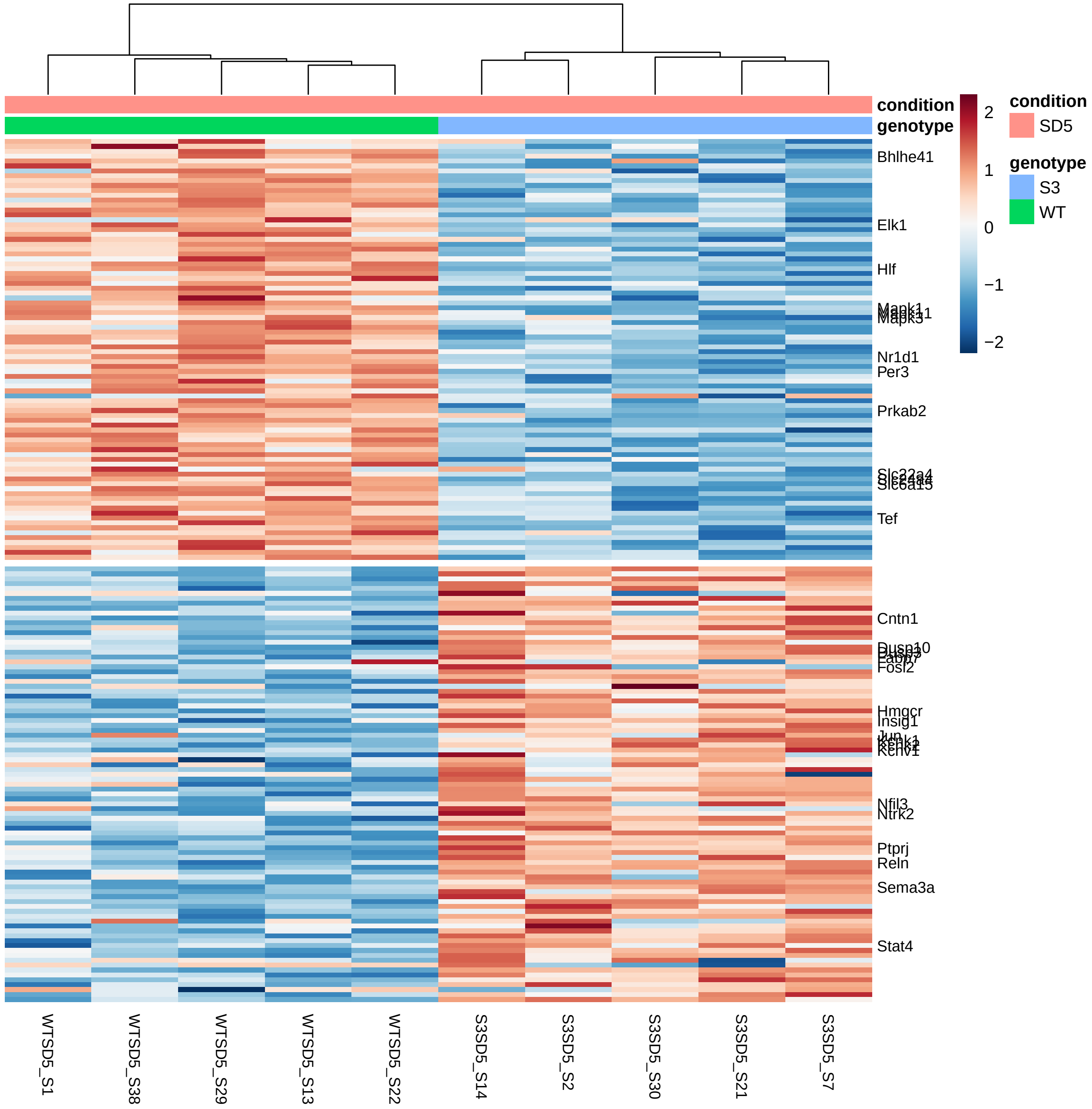


```
save_pheatmap_pdf(filename="plots/heatmap_gg_sd_only.pdf", plot=ph1, width=20, height=20)
```

```
## pdf
##   2
```

Group gene profiles

Group gene profiles by genotype

```
g <- geneGroupProfileRows(normalized.counts=normExprData, design.matrix=designMatrix,
            gene.names=c("Nr1d1", "Hlf", "Per3", "Bhlhe41", "Tef"),
            res.o=de.genes.symb, show.plot=TRUE, plotly.flag=FALSE, log.flag=TRUE)
print(g)
```

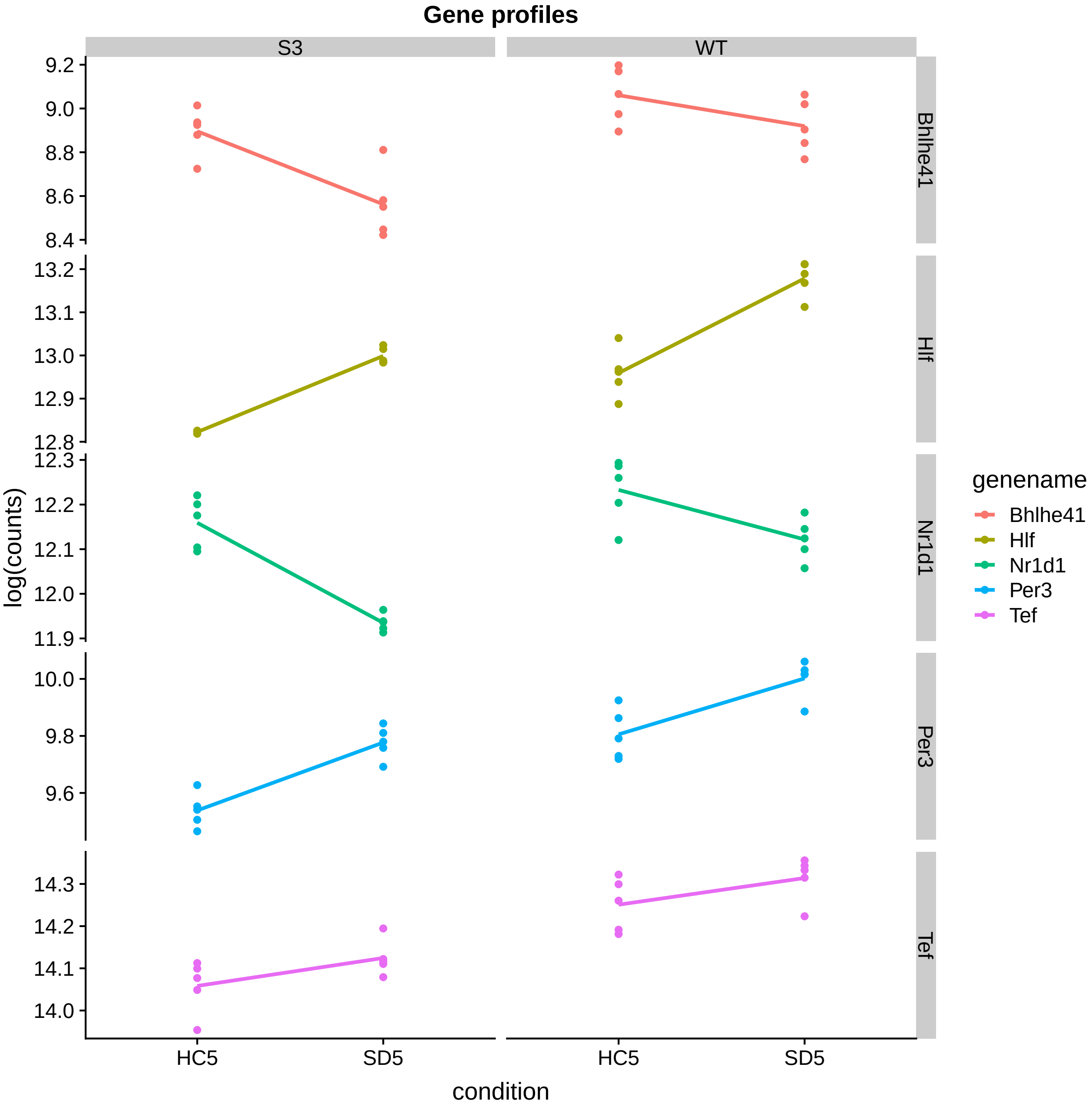


```
save_plot(filename=paste0("plots/", "Nr1d1_Hlf_Per3_Bhlhe41_Tef", "_log_gene_profile_genotype.pdf"), pl
        base_height=15, base_width=15)
```

Group gene profiles by condition

```
g <- geneGroupProfileRowsRev(normalized.counts=normExprData, design.matrix=designMatrix,
            gene.names=c("Nr1d1", "Hlf", "Per3", "Bhlhe41", "Tef"),
            res.o=de.genes.symb, show.plot=TRUE, plotly.flag=FALSE, log.flag=TRUE)
print(g)
```

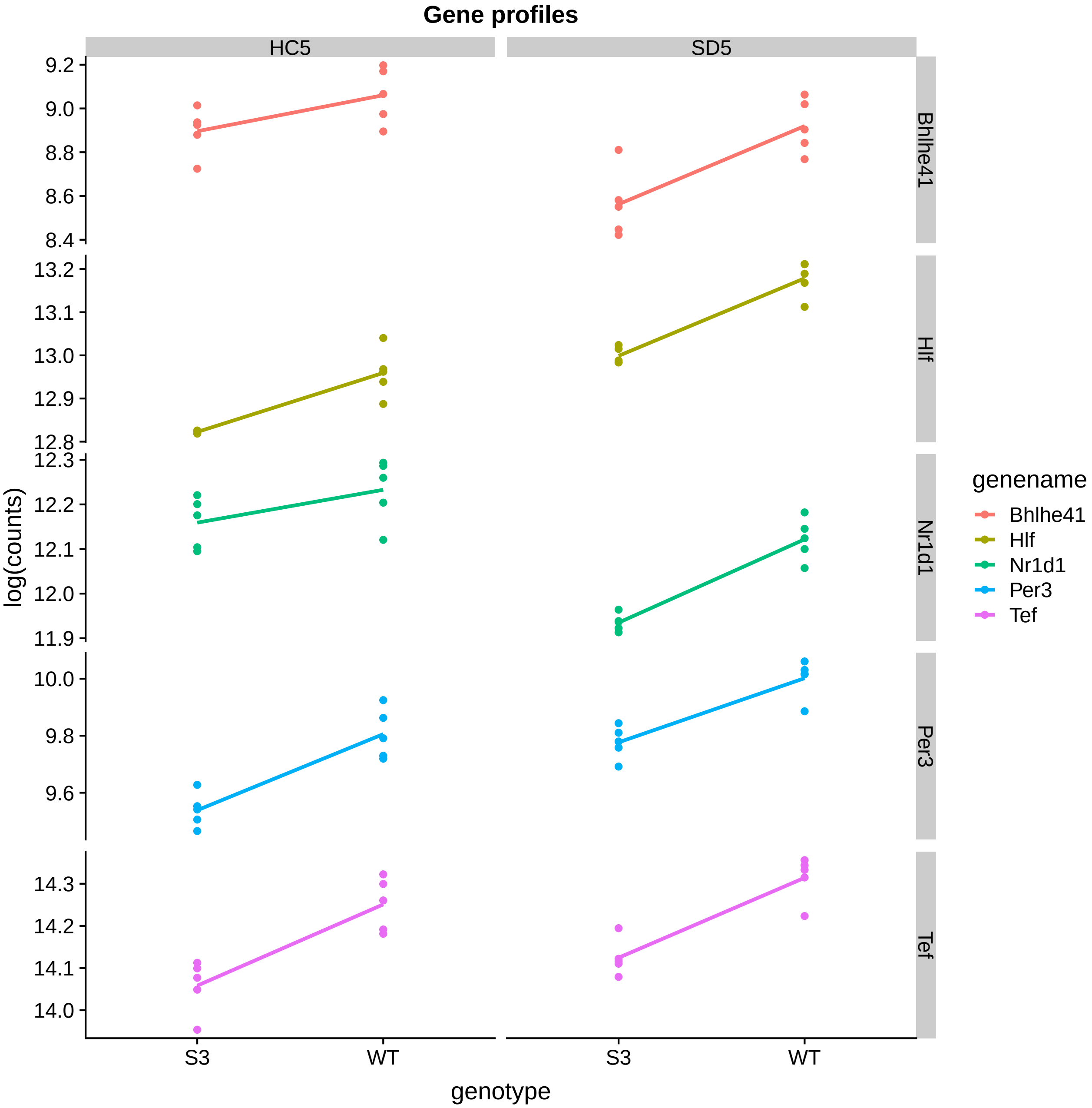


```
save_plot(filename=paste0("plots/", "Nr1d1_Hlf_Per3_Bhlhe41_Tef", "_log_gene_profile_condition.pdf"), pl
        base_height=15, base_width=15)
```

Circadian Analysis LD-DD

Analysis for activity

```
wt <- read_xlsx("data/LD_DD_Activity_analysis_4_R.xlsx", sheet = 1)
mut <- read_xlsx("data/LD_DD_Activity_analysis_4_R.xlsx", sheet = 2)

wt <- wt %>%
  bind_cols(WT.M=rep("WT", nrow(wt)), time = decimal_date(ymd(wt$`Total_revolutions/day`)), .) %>%
```

```r
  gather(mice, activity, -c(1:5))  %>%
  mutate(time = time-min(time)) %>%
  dplyr::select(-`Total_revolutions/day`)

mut <- mut %>%
  bind_cols(WT.M=rep("M", nrow(mut)), time = decimal_date(ymd(mut$`Total_revolutions/day`)), .) %>%
  gather(mice, activity, -c(1:5))  %>%
  mutate(time = time-min(time)) %>%
  dplyr::select(-`Total_revolutions/day`)

data <- wt %>% bind_rows(mut)

data <- data %>% filter(week>=3)

data$mice <- factor(data$mice, levels= unique(data$mice))
data$time_scaled <- scale(data$time, scale=FALSE)
data$period <- factor(data$period, levels= unique(data$period))
data$WT.M <-factor(data$WT.M, levels=c("WT", "M"))

mod <- lme(activity ~ time_scaled * WT.M, random=~1|mice, data = data)

cat("Estimates, errors and the significance")
```

```
## Estimates, errors and the significance
```

```r
summary(mod)
```

```
## Linear mixed-effects model fit by REML
##  Data: data
##        AIC      BIC   logLik
##   8681.339 8705.303 -4334.67
##
## Random effects:
##  Formula: ~1 | mice
##         (Intercept) Residual
## StdDev:    14936.81 11161.46
##
## Fixed effects: activity ~ time_scaled * WT.M
##                       Value Std.Error  DF   t-value p-value
## (Intercept)         38778.45   5335.29 388  7.268296  0.0000
## time_scaled        -20486.52  35588.68 388 -0.575647  0.5652
## WT.MM              -20324.26   7810.06  13 -2.602317  0.0219
## time_scaled:WT.MM -289880.69  52096.49 388 -5.564304  0.0000
##  Correlation:
##                   (Intr) tm_scl WT.MM
## time_scaled        0.000
## WT.MM             -0.683  0.000
## time_scaled:WT.MM  0.000 -0.683  0.000
##
## Standardized Within-Group Residuals:
##         Min          Q1         Med          Q3         Max
## -2.69133816 -0.63470177  0.03277689  0.63109234  3.19701990
##
## Number of Observations: 405
```

```
## Number of Groups: 15
```

```r
cat("Bootstrap confidence intervals for the estimates")
```

```
## Bootstrap confidence intervals for the estimates
```

```r
mod_lmer <- lmer(activity ~ time_scaled * WT.M + (1|mice), data = data)
suppressMessages(confint.merMod(mod_lmer, method = "boot", nsim = 999))
```

```
##                          2.5 %      97.5 %
## .sig01                8955.955   20503.836
## .sigma               10389.639   12032.640
## (Intercept)          28479.152   48421.285
## time_scaled         -87582.692   48954.981
## WT.MM               -36065.660   -4532.535
## time_scaled:WT.MM -384418.156 -187596.736
```

```r
cat("ANOVA table")
```

```
## ANOVA table
```

```r
anova.lme(mod, type = "marginal", adjustSigma = F)
```

```
##                  numDF denDF  F-value p-value
## (Intercept)          1   388 52.82812  <.0001
## time_scaled          1   388  0.33137  0.5652
## WT.M                 1    13  6.77206  0.0219
## time_scaled:WT.M     1   388 30.96148  <.0001
```

Analysis for alpha

```r
wt <- read_xlsx("data/LD_DD_Alpha_Activity_analysis_4_R.xlsx", sheet = 1, na = "NA")
mut <- read_xlsx("data/LD_DD_Alpha_Activity_analysis_4_R.xlsx", sheet = 2, na = "NA")

wt <- wt %>%
  bind_cols(WT.M = rep("WT", nrow(wt)), time = decimal_date(ymd(wt$`Total_revolutions/day`)), .)%>%
  gather(mice, alpha, -c(1:5))  %>%
  mutate(time = time-min(time)) %>%
  dplyr::select(-`Total_revolutions/day`)

mut <- mut %>%
  bind_cols(WT.M=rep("M", nrow(mut)), time = decimal_date(ymd(mut$`Total_revolutions/day`)), .)%>%
  gather(mice, alpha, -c(1:5))  %>%
  mutate(time = time-min(time)) %>%
  dplyr::select(-`Total_revolutions/day`)

alpha_data <- wt %>% bind_rows(mut)

alpha_data <- alpha_data %>% filter(week>=3)
alpha_data<- na.omit(alpha_data)

alpha_data$mice <- factor(alpha_data$mice, levels= unique(alpha_data$mice))
alpha_data$time_scaled <- scale(alpha_data$time, scale=FALSE)
alpha_data$period <- factor(alpha_data$period, levels= unique(alpha_data$period))
alpha_data$WT.M <- factor(alpha_data$WT.M, levels=c("WT", "M"))
alpha_data$alpha <-  as.numeric(alpha_data$alpha)
```

```r
mod1 <- lme(alpha ~ time_scaled * WT.M, random=~1|mice, data = alpha_data, na.action = na.omit)

cat("Estimates, errors and the significance")
```

```
## Estimates, errors and the significance
```

```r
summary(mod1)
```

```
## Linear mixed-effects model fit by REML
##  Data: alpha_data
##        AIC      BIC    logLik
##   2068.243 2091.978 -1028.121
##
## Random effects:
##  Formula: ~1 | mice
##         (Intercept) Residual
## StdDev:   0.6720236 3.405597
##
## Fixed effects: alpha ~ time_scaled * WT.M
##                          Value Std.Error  DF   t-value p-value
## (Intercept)        10.101010  0.334981 373 30.154013  0.0000
## time_scaled       -16.267322 11.031558 373 -1.474617  0.1412
## WT.MM              -0.714526  0.490361  13 -1.457142  0.1688
## time_scaled:WT.MM   2.360361 16.148547 373  0.146166  0.8839
##  Correlation:
##                   (Intr) tm_scl WT.MM
## time_scaled        0.000
## WT.MM             -0.683  0.000
## time_scaled:WT.MM  0.000 -0.683  0.000
##
## Standardized Within-Group Residuals:
##         Min          Q1         Med          Q3         Max
## -2.73631345 -0.48276146  0.06646042  0.49709145  3.94949030
##
## Number of Observations: 390
## Number of Groups: 15
```

```r
cat("Bootstrap confidence intervals for the estimates")
```

```
## Bootstrap confidence intervals for the estimates
```

```r
mod1_lmer <- lmer(alpha ~ time_scaled * WT.M + (1|mice), data = alpha_data)
suppressMessages(confint.merMod(mod1_lmer, method = "boot", nsim = 999))
```

```
##                         2.5 %    97.5 %
## .sig01              0.000000  1.126554
## .sigma              3.147126  3.655970
## (Intercept)         9.461287 10.763733
## time_scaled       -38.593893  4.970986
## WT.MM              -1.680165  0.235239
## time_scaled:WT.MM -29.410802 34.100068
```

```r
cat("ANOVA table")
```

```
## ANOVA table
```

```r
anova.lme(mod1, type = "marginal", adjustSigma = F)
```

```
##                    numDF denDF  F-value p-value
## (Intercept)            1   373 909.2645  <.0001
## time_scaled            1   373   2.1745  0.1412
## WT.M                   1    13   2.1233  0.1688
## time_scaled:WT.M       1   373   0.0214  0.8839
```

Analysis for period

```r
wt <- read_xlsx("data/LD_DD_Period_analysis_4_R.xlsx", sheet = 1)  %>% gather(mice, value, -1)
wt <- data.frame(WT.M=rep("WT", nrow(wt))) %>% bind_cols(wt)
mut <- read_xlsx("data/LD_DD_Period_analysis_4_R.xlsx", sheet = 2)  %>% gather(mice, value, -1)
mut <- data.frame(WT.M=rep("M", nrow(mut))) %>% bind_cols(mut)

period_data <- wt %>% bind_rows(mut)
period_data$value <- as.numeric(period_data$value)

mod2 <- lme(value~ week * WT.M, random = ~1|mice, data = period_data)

cat("Estimates, errors and the significance")
```

```
## Estimates, errors and the significance
```

```r
summary(mod2)
```

```
## Linear mixed-effects model fit by REML
##  Data: period_data
##         AIC   BIC    logLik
##   309.1875 328.7 -144.5938
##
## Random effects:
##  Formula: ~1 | mice
##         (Intercept) Residual
## StdDev:   0.7251103 3.268825
##
## Fixed effects: value ~ week * WT.M
##                          Value Std.Error DF   t-value p-value
## (Intercept)          23.721429  1.265532 39 18.744234  0.0000
## weekDD_Week_2        -3.381429  1.747260 39 -1.935275  0.0602
## weekDD_Week_3         2.055714  1.747260 39  1.176536  0.2465
## weekLD_Week_3         0.208571  1.747260 39  0.119371  0.9056
## WT.MWT                0.137321  1.732901 13  0.079244  0.9380
## weekDD_Week_2:WT.MWT  3.251429  2.392535 39  1.358989  0.1820
## weekDD_Week_3:WT.MWT -2.426964  2.392535 39 -1.014390  0.3166
## weekLD_Week_3:WT.MWT -0.063571  2.392535 39 -0.026571  0.9789
##  Correlation:
##                      (Intr) wkDD_W_2 wkDD_W_3 wkLD_W_3 WT.MWT wDD_W_2:
## weekDD_Week_2        -0.690
## weekDD_Week_3        -0.690  0.500
## weekLD_Week_3        -0.690  0.500    0.500
## WT.MWT               -0.730  0.504    0.504    0.504
## weekDD_Week_2:WT.MWT  0.504 -0.730   -0.365   -0.365   -0.690
## weekDD_Week_3:WT.MWT  0.504 -0.365   -0.730   -0.365   -0.690  0.500
```

```
## weekLD_Week_3:WT.MWT  0.504 -0.365   -0.365   -0.730   -0.690  0.500
##                       wDD_W_3:
## weekDD_Week_2
## weekDD_Week_3
## weekLD_Week_3
## WT.MWT
## weekDD_Week_2:WT.MWT
## weekDD_Week_3:WT.MWT
## weekLD_Week_3:WT.MWT  0.500
##
## Standardized Within-Group Residuals:
##          Min           Q1          Med           Q3          Max
## -5.938352181 -0.067963537 -0.001414478  0.093674882  1.778532447
##
## Number of Observations: 60
## Number of Groups: 15
```

```
cat("Bootstrap confidence intervals for the estimates")
```

```
## Bootstrap confidence intervals for the estimates
```

```
mod2_lmer <- lmer(value ~ week * WT.M + (1|mice), data = period_data)
suppressMessages(confint.merMod(mod2_lmer, method = "boot", nsim = 999))
```

```
##                           2.5 %     97.5 %
## .sig01                 0.000000  2.1431666
## .sigma                 2.513939  3.8493530
## (Intercept)           21.295015 26.0826407
## weekDD_Week_2         -6.777347  0.0616212
## weekDD_Week_3         -1.202368  5.3482622
## weekLD_Week_3         -3.176618  3.6860414
## WT.MWT                -3.217573  3.2653549
## weekDD_Week_2:WT.MWT -1.222346  8.1648739
## weekDD_Week_3:WT.MWT -6.663716  1.9294315
## weekLD_Week_3:WT.MWT -4.658114  4.2191458
```

```
cat("ANOVA table")
```

```
## ANOVA table
```

```
anova.lme(mod2, type = "marginal", adjustSigma = F)
```

```
##             numDF denDF  F-value p-value
## (Intercept)     1    39 351.3463  <.0001
## week            3    39   3.3611  0.0282
## WT.M            1    13   0.0063  0.9380
## week:WT.M       3    39   1.9008  0.1454
```

Circadian Analysis LD-LD

Analysis for activity

```
wt <- read_xlsx("data/LD_LD_Activity_analysis_4_R.xlsx", sheet = 1)
mut <- read_xlsx("data/LD_LD_Activity_analysis_4_R.xlsx", sheet = 2)

wt <- wt %>%
```

```
  bind_cols(WT.M=rep("WT", nrow(wt)), time = decimal_date(ymd(wt$`Total_revolutions/day`)), .) %>%
  gather(mice, activity, -c(1:3))  %>%
  mutate(time = time-min(time)) %>%
  dplyr::select(-`Total_revolutions/day`)

mut <- mut %>%
  bind_cols(WT.M=rep("M", nrow(mut)), time = decimal_date(ymd(mut$`Total_revolutions/day`)), .) %>%
  gather(mice, activity, -c(1:3))  %>%
  mutate(time = time-min(time)) %>%
  dplyr::select(-`Total_revolutions/day`)

data <- wt %>% bind_rows(mut)


data$mice <- factor(data$mice, levels= unique(data$mice))
data$time_scaled <- scale(data$time, scale=FALSE)
data$WT.M <-factor(data$WT.M, levels=c("WT", "M"))

mod3 <- lme(activity ~ time_scaled * WT.M, random=~1|mice, data = data)

cat("Estimates, errors and the significance")
```

```
## Estimates, errors and the significance
```

```
summary(mod3)
```

```
## Linear mixed-effects model fit by REML
##  Data: data
##        AIC      BIC    logLik
##   11661.69 11687.61 -5824.844
##
## Random effects:
##  Formula: ~1 | mice
##         (Intercept) Residual
## StdDev:    14529.36 7940.975
##
## Fixed effects: activity ~ time_scaled * WT.M
##                      Value Std.Error  DF   t-value p-value
## (Intercept)       40118.57  5158.779 542  7.776758  0.0000
## time_scaled        2089.01  1016.983 542  2.054126  0.0404
## WT.MM            -19109.48  7295.615  14 -2.619311  0.0202
## time_scaled:WT.MM -13336.51  1438.232 542 -9.272853  0.0000
##  Correlation:
##                   (Intr) tm_scl WT.MM
## time_scaled        0.000
## WT.MM             -0.707  0.000
## time_scaled:WT.MM  0.000 -0.707  0.000
##
## Standardized Within-Group Residuals:
##         Min          Q1         Med          Q3         Max
## -3.44738653 -0.64119095  0.03137236  0.52237000  3.77912348
##
## Number of Observations: 560
## Number of Groups: 16
```

```r
cat("Bootstrap confidence intervals for the estimates")
```

```
## Bootstrap confidence intervals for the estimates
```

```r
mod_lmer3 <- lmer(activity ~ time_scaled * WT.M + (1|mice), data = data)
suppressMessages(confint.merMod(mod_lmer3, method = "boot", nsim = 999))
```

```
##                          2.5 %     97.5 %
## .sig01              8901.99617  19858.047
## .sigma              7467.25075   8430.771
## (Intercept)        29846.58217  50229.607
## time_scaled           79.33709   4227.370
## WT.MM             -33900.29899  -5397.748
## time_scaled:WT.MM -16350.14572 -10643.563
```

```r
cat("ANOVA table")
```

```
## ANOVA table
```

```r
anova.lme(mod3, type = "marginal", adjustSigma = F)
```

```
##                  numDF denDF  F-value p-value
## (Intercept)          1   542 60.47796  <.0001
## time_scaled          1   542  4.21943  0.0404
## WT.M                 1    14  6.86079  0.0202
## time_scaled:WT.M     1   542 85.98580  <.0001
```

Analysis for alpha

```r
wt <- read_xlsx("data/LD_LD_Alpha_Activity_analysis_4_R.xlsx", sheet = 1, na = "NA")
mut <- read_xlsx("data/LD_LD_Alpha_Activity_analysis_4_R.xlsx", sheet = 2, na = "NA")

wt <- wt %>%
  bind_cols(WT.M = rep("WT", nrow(wt)), time = decimal_date(ymd(wt$`Total_revolutions/day`)), .)%>%
  gather(mice, alpha, -c(1:3))  %>%
  mutate(time = time-min(time)) %>%
  dplyr::select(-`Total_revolutions/day`)

mut <- mut %>%
  bind_cols(WT.M=rep("M", nrow(mut)), time = decimal_date(ymd(mut$`Total_revolutions/day`)), .)%>%
  gather(mice, alpha, -c(1:3))  %>%
  mutate(time = time-min(time)) %>%
  dplyr::select(-`Total_revolutions/day`)

alpha_data <- wt %>% bind_rows(mut)

alpha_data<- na.omit(alpha_data)

alpha_data$mice <- factor(alpha_data$mice, levels= unique(alpha_data$mice))
alpha_data$time_scaled <- scale(alpha_data$time, scale=FALSE)
alpha_data$WT.M <- factor(alpha_data$WT.M, levels=c("WT", "M"))
alpha_data$alpha <-  as.numeric(alpha_data$alpha)

mod4 <- lme(alpha ~ time_scaled * WT.M, random=~1|mice, data = alpha_data, na.action = na.omit)

cat("Estimates, errors and the significance")
```

```
## Estimates, errors and the significance
```

```r
summary(mod4)
```

```
## Linear mixed-effects model fit by REML
##   Data: alpha_data
##        AIC     BIC    logLik
##   3188.158 3214.58 -1588.079
##
## Random effects:
##  Formula: ~1 | mice
##         (Intercept) Residual
## StdDev:    1.301648 3.255454
##
## Fixed effects: alpha ~ time_scaled * WT.M
##                          Value Std.Error  DF   t-value p-value
## (Intercept)          10.090329  0.496636 590 20.317334  0.0000
## time_scaled         -18.117603  6.214771 590 -2.915249  0.0037
## WT.MM                -1.255362  0.702350  14 -1.787374  0.0955
## time_scaled:WT.MM     6.997015  8.789013 590  0.796109  0.4263
##  Correlation:
##                   (Intr) tm_scl WT.MM
## time_scaled        0.000
## WT.MM             -0.707  0.000
## time_scaled:WT.MM  0.000 -0.707  0.000
##
## Standardized Within-Group Residuals:
##         Min          Q1         Med          Q3         Max
## -2.87208043 -0.48234294 -0.01803405  0.36708937  4.80926449
##
## Number of Observations: 608
## Number of Groups: 16
```

```r
cat("Bootstrap confidence intervals for the estimates")
```

```
## Bootstrap confidence intervals for the estimates
```

```r
mod4_lmer <- lmer(alpha ~ time_scaled * WT.M + (1|mice), data = alpha_data)
suppressMessages(confint.merMod(mod4_lmer, method = "boot", nsim = 999))
```

```
##                         2.5 %     97.5 %
## .sig01              0.6836094  1.8671868
## .sigma              3.0747216  3.4248900
## (Intercept)         9.0862073 11.0284163
## time_scaled       -29.7653210 -5.7385238
## WT.MM              -2.6644755  0.1792939
## time_scaled:WT.MM -10.6214446 23.6632498
```

```r
cat("ANOVA table")
```

```
## ANOVA table
```

```r
anova.lme(mod4, type = "marginal", adjustSigma = F)
```

```
##                   numDF denDF  F-value p-value
## (Intercept)           1   590 412.7941  <.0001
```

```
## time_scaled          1   590   8.4987  0.0037
## WT.M                 1    14   3.1947  0.0955
## time_scaled:WT.M     1   590   0.6338  0.4263
```

Analysis for period

```
wt <- read_xlsx("data/LD_LD_Period_analysis_4_R.xlsx", sheet = 1)  %>% gather(mice, value, -1)
wt <- data.frame(WT.M=rep("WT", nrow(wt))) %>% bind_cols(wt)
mut <- read_xlsx("data/LD_LD_Period_analysis_4_R.xlsx", sheet = 2)  %>% gather(mice, value, -1)
mut <- data.frame(WT.M=rep("M", nrow(mut))) %>% bind_cols(mut)

period_data <- wt %>% bind_rows(mut)
period_data$value <- as.numeric(period_data$value)

mod5 <- lme(value ~ week * WT.M, random = ~1|mice, data = period_data)

cat("Estimates, errors and the significance")
```

```
## Estimates, errors and the significance
```

```
summary(mod5)
```

```
## Linear mixed-effects model fit by REML
##  Data: period_data
##        AIC      BIC    logLik
##   373.0096 399.9916 -174.5048
##
## Random effects:
##  Formula: ~1 | mice
##         (Intercept) Residual
## StdDev:   0.3722568 2.496563
##
## Fixed effects: value ~ week * WT.M
##                           Value Std.Error DF   t-value p-value
## (Intercept)            21.26375 0.8924266 56 23.826890  0.0000
## weekLD_Week_2           2.59000 1.2482815 56  2.074853  0.0426
## weekLD_Week_3           2.98500 1.2482815 56  2.391288  0.0202
## weekLD_Week_4           2.75500 1.2482815 56  2.207034  0.0314
## weekLD_Week_5           2.81000 1.2482815 56  2.251095  0.0283
## WT.MWT                  2.75375 1.2620818 14  2.181911  0.0467
## weekLD_Week_2:WT.MWT -2.62000 1.7653366 56 -1.484136  0.1434
## weekLD_Week_3:WT.MWT -3.02375 1.7653366 56 -1.712846  0.0923
## weekLD_Week_4:WT.MWT -2.76125 1.7653366 56 -1.564149  0.1234
## weekLD_Week_5:WT.MWT -2.81625 1.7653366 56 -1.595305  0.1163
##  Correlation:
##                      (Intr) wkLD_W_2 wkLD_W_3 wkLD_W_4 wkLD_W_5 WT.MWT
## weekLD_Week_2        -0.699
## weekLD_Week_3        -0.699  0.500
## weekLD_Week_4        -0.699  0.500    0.500
## weekLD_Week_5        -0.699  0.500    0.500    0.500
## WT.MWT               -0.707  0.495    0.495    0.495    0.495
## weekLD_Week_2:WT.MWT  0.495 -0.707   -0.354   -0.354   -0.354   -0.699
## weekLD_Week_3:WT.MWT  0.495 -0.354   -0.707   -0.354   -0.354   -0.699
## weekLD_Week_4:WT.MWT  0.495 -0.354   -0.354   -0.707   -0.354   -0.699
## weekLD_Week_5:WT.MWT  0.495 -0.354   -0.354   -0.354   -0.707   -0.699
```

```
##                         wLD_W_2: wLD_W_3: wLD_W_4:
## weekLD_Week_2
## weekLD_Week_3
## weekLD_Week_4
## weekLD_Week_5
## WT.MWT
## weekLD_Week_2:WT.MWT
## weekLD_Week_3:WT.MWT  0.500
## weekLD_Week_4:WT.MWT  0.500    0.500
## weekLD_Week_5:WT.MWT  0.500    0.500    0.500
##
## Standardized Within-Group Residuals:
##          Min           Q1          Med           Q3          Max
## -7.277846353 -0.022845832 -0.004456104  0.031513974  2.241303977
##
## Number of Observations: 80
## Number of Groups: 16
```

```
cat("Bootstrap confidence intervals for the estimates")
```

```
## Bootstrap confidence intervals for the estimates
```

```
mod5_lmer <- lmer(value ~ week * WT.M + (1|mice), data = period_data)
suppressMessages(confint.merMod(mod5_lmer, method = "boot", nsim = 999))
```

```
##                              2.5 %     97.5 %
## .sig01                 0.000000000  1.3313374
## .sigma                 2.001288877  2.8729869
## (Intercept)           19.496530977 23.0898564
## weekLD_Week_2          0.006424996  5.1844249
## weekLD_Week_3          0.496212606  5.3496704
## weekLD_Week_4          0.238413883  5.1431180
## weekLD_Week_5          0.174869466  5.2394188
## WT.MWT                 0.263083364  5.1512889
## weekLD_Week_2:WT.MWT -5.995340021  0.8855984
## weekLD_Week_3:WT.MWT -6.395424170  0.3820959
## weekLD_Week_4:WT.MWT -6.296576299  0.7338638
## weekLD_Week_5:WT.MWT -6.202013468  0.6590960
```

```
cat("ANOVA table")
```

```
## ANOVA table
```

```
anova.lme(mod5, type = "marginal", adjustSigma = F)
```

```
##             numDF denDF  F-value p-value
## (Intercept)     1    56 567.7207  <.0001
## week            4    56   2.0166  0.1045
## WT.M            1    14   4.7607  0.0467
## week:WT.M       4    56   1.0236  0.4031
```

Analysis for Spectral Data

```
# GAM plots
library(mgcv)
```

```r
data<-read_xlsx("data/BL_spectral.xlsx")

data <- data %>% gather(Hertz, value,  -c(1:3))
data <- data %>%
  mutate(GT=replace(GT,GT == 1, "WT")) %>%
  mutate(GT=replace(GT,GT == 2, "MT")) %>%
  mutate(LD=replace(LD,LD == 1, "LIGHT")) %>%
  mutate(LD=replace(LD,LD == 2, "DARK")) %>%
  mutate(hz= as.numeric(Hertz)) %>%
  mutate(STATE = factor(STATE, levels = unique(STATE))) %>%
  mutate(GT = factor(GT, levels = unique(GT))) %>%
  mutate(LD  = factor(LD, levels = unique(LD))) %>%
  mutate(value = replace(value,value == -99, NA))

temp<-data %>% filter(STATE == "WAKEFULNESS" & LD == "LIGHT")
index <-paste(data$STATE, data$LD, sep = "")
index_lev <- unique(index)

layout(matrix(seq_len(6), nrow = 3, ncol = 2, byrow = TRUE))
shadow_col <- c(rgb(109, 109, 109, max = 255, alpha = 80),
                rgb(244, 66, 66, max = 255, alpha = 80))

for(this_index in index_lev) {
  state <- unique(data[index == this_index, ][, 1])
  state <- as.character(unlist(state))
  light <- unique(data[index == this_index, ][, 3])
  light <- as.character(unlist(light))

  temp2 <- data[index == this_index, ]
  plot(x = temp2$hz, y = temp2$value, type = "n",
       ylab =  "% of Total Power", ylim = c(0,20),
       xlab = 'Hertz', lwd = 3, cex = 1.2,
       main = paste0(state, "-", light), axes = FALSE)
  axis(1, at = seq(0, 20, by = 5), las = 1, pos = 0, lwd = 3)
  axis(2, at = seq(0, 15, by = 3), las = 2, pos = 0, lwd = 3)

  mod <- list(wt = gam(value~s(hz), data = temp2[temp2$GT == "WT",]),
              mt = gam(value~s(hz), data = temp2[temp2$GT == "MT",]))

  for(i in seq_along(mod)) {
    ss <- seq(min(temp2$hz) + 0.1, max(temp2$hz) - 0.1, 0.1)
    pred <- predict(mod[[i]], data.frame(hz = ss), se = TRUE)
    fit <- pred$fit
    se <- pred$se.fit
    lower <- fit - 1.96 * se
    upper  <- fit + 1.96 * se
    to_plot <- data.frame(hz = ss, fit, lower, upper)

    polygon(c(to_plot$hz, rev(to_plot$hz)),
            c(to_plot$lower, rev(to_plot$upper)),
            col = shadow_col[i],
            border = NA)
```

```
    lines(to_plot$hz, fit,  lwd=2, col = c("black", "red")[i])
  }
  legend(x = "topright",horiz = TRUE, c("MT", "WT"),
         pch = c(NA, NA), col = c("black", "red"),
         inset = c(0, -0.08), cex = 1,  bty = 'n',
         box.lty = c('NA', 'NA'), lwd = 3 )

}
```

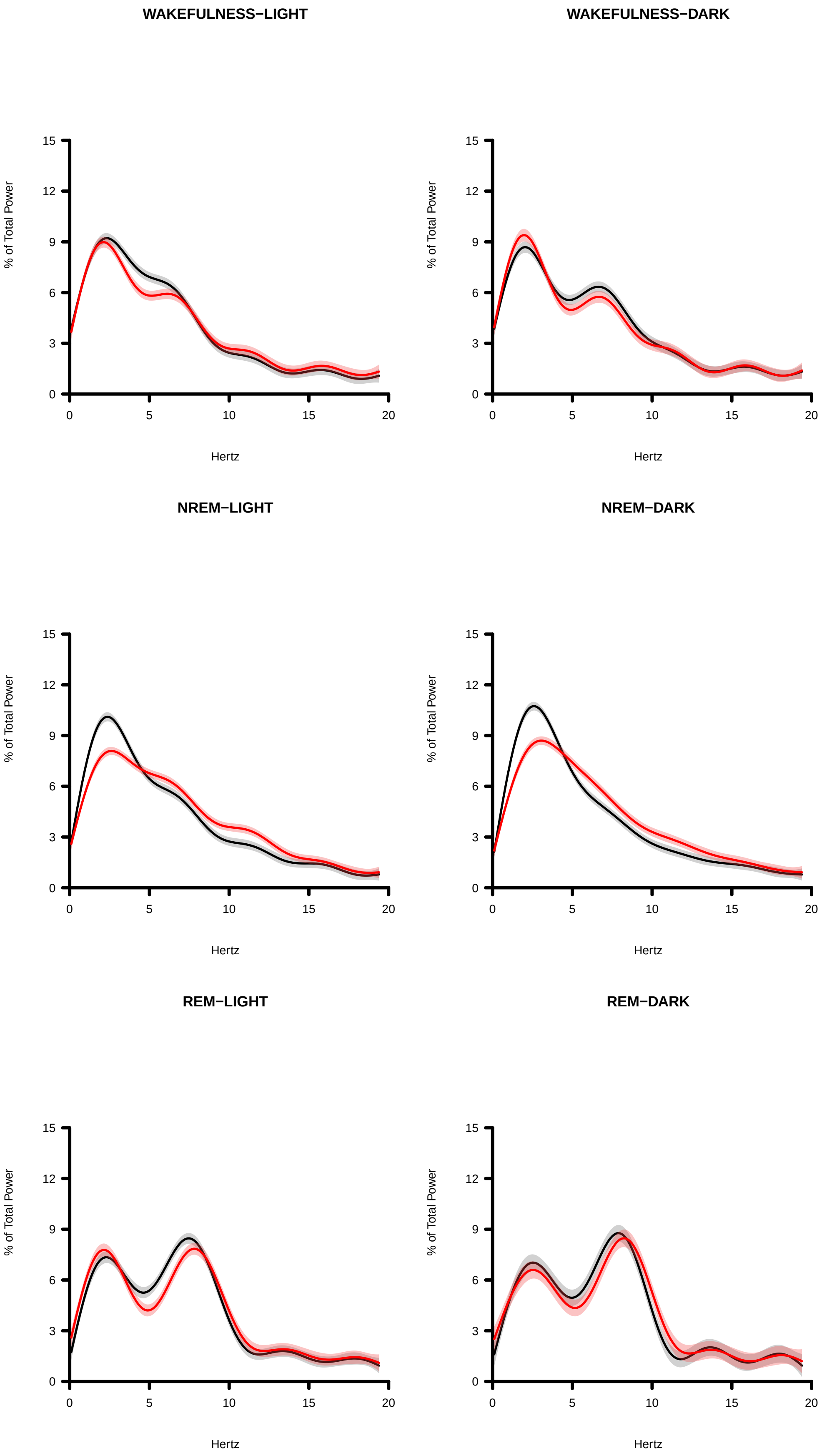
WAKEFULNESS−LIGHT
WAKEFULNESS−DARK
NREM−LIGHT
NREM−DARK
REM−LIGHT
REM−DARK
% of Total Power
Hertz
0
3
6
9
12
15
0
5
10
15
20

Session Info

```r
sessionInfo()
```

```
## R version 3.5.2 (2018-12-20)
## Platform: x86_64-pc-linux-gnu (64-bit)
## Running under: Arch Linux
##
## Matrix products: default
## BLAS: /usr/lib/libblas.so.3.8.0
## LAPACK: /usr/lib/liblapack.so.3.8.0
##
## locale:
##  [1] LC_CTYPE=en_US.UTF-8       LC_NUMERIC=C
##  [3] LC_TIME=en_US.UTF-8        LC_COLLATE=en_US.UTF-8
##  [5] LC_MONETARY=en_US.UTF-8    LC_MESSAGES=en_US.UTF-8
##  [7] LC_PAPER=en_US.UTF-8       LC_NAME=C
##  [9] LC_ADDRESS=C               LC_TELEPHONE=C
## [11] LC_MEASUREMENT=en_US.UTF-8 LC_IDENTIFICATION=C
##
## attached base packages:
## [1] stats4    parallel  stats     graphics  grDevices utils     datasets
## [8] methods   base
##
## other attached packages:
##  [1] mgcv_1.8-27                 dendextend_1.9.0
##  [3] pheatmap_1.0.12             clusterExperiment_2.2.0
##  [5] SingleCellExperiment_1.4.1  gplots_3.0.1.1
##  [7] plyr_1.8.4                  org.Mm.eg.db_3.7.0
##  [9] AnnotationDbi_1.44.0        readxl_1.3.0
## [11] lme4_1.1-20                 Matrix_1.2-15
## [13] nlme_3.1-137                tidyr_0.8.2
## [15] dplyr_0.8.0.1               lubridate_1.7.4
## [17] cowplot_0.9.4               RUVSeq_1.16.1
## [19] edgeR_3.24.3                limma_3.38.3
## [21] EDASeq_2.16.3               ShortRead_1.40.0
## [23] GenomicAlignments_1.18.1    SummarizedExperiment_1.12.0
## [25] DelayedArray_0.8.0          matrixStats_0.54.0
## [27] Rsamtools_1.34.1            GenomicRanges_1.34.0
## [29] GenomeInfoDb_1.18.2         Biostrings_2.50.2
## [31] XVector_0.22.0              IRanges_2.16.0
## [33] S4Vectors_0.20.1            BiocParallel_1.16.6
## [35] Biobase_2.42.0              BiocGenerics_0.28.0
## [37] plotly_4.8.0                ggplot2_3.1.0
##
## loaded via a namespace (and not attached):
##   [1] copula_0.999-19       uuid_0.1-2            aroma.light_3.12.0
##   [4] NMF_0.21.0            lazyeval_0.2.1        splines_3.5.2
##   [7] pspline_1.0-18        rncl_0.8.3            NOISeq_2.26.1
##  [10] gridBase_0.4-7        digest_0.6.18         foreach_1.4.4
##  [13] htmltools_0.3.6       viridis_0.5.1         gdata_2.18.0
##  [16] magrittr_1.5          memoise_1.1.0         cluster_2.0.7-1
##  [19] doParallel_1.0.14     annotate_1.60.0       stabledist_0.7-1
##  [22] R.utils_2.8.0         prettyunits_1.0.2     colorspace_1.4-0
```

```
##  [25] blob_1.1.1             xfun_0.5               crayon_1.3.4
##  [28] RCurl_1.95-4.11        jsonlite_1.6           genefilter_1.64.0
##  [31] phylobase_0.8.6        survival_2.43-3        iterators_1.0.10
##  [34] ape_5.2                glue_1.3.0             registry_0.5
##  [37] gtable_0.2.0           zlibbioc_1.28.0        kernlab_0.9-27
##  [40] Rhdf5lib_1.4.2         HDF5Array_1.10.1       DEoptimR_1.0-8
##  [43] prabclus_2.2-7         scales_1.0.0           DESeq_1.34.1
##  [46] mvtnorm_1.0-8          DBI_1.0.0              rngtools_1.3.1
##  [49] bibtex_0.4.2           Rcpp_1.0.0             viridisLite_0.3.0
##  [52] xtable_1.8-3           progress_1.2.0         bit_1.1-14
##  [55] mclust_5.4.2           glmnet_2.0-16          htmlwidgets_1.3
##  [58] httr_1.4.0             RColorBrewer_1.1-2     fpc_2.1-11.1
##  [61] modeltools_0.2-22      pkgconfig_2.0.2        XML_3.98-1.17
##  [64] R.methodsS3_1.7.1      flexmix_2.3-15         nnet_7.3-12
##  [67] locfit_1.5-9.1         softImpute_1.4         howmany_0.3-1
##  [70] tidyselect_0.2.5       labeling_0.3           rlang_0.3.1
##  [73] reshape2_1.4.3         munsell_0.5.0          cellranger_1.1.0
##  [76] tools_3.5.2            cli_1.0.1              RSQLite_2.1.1
##  [79] ade4_1.7-13            evaluate_0.13          stringr_1.4.0
##  [82] yaml_2.2.0             knitr_1.21             bit64_0.9-7
##  [85] robustbase_0.93-3      caTools_1.17.1.1       purrr_0.3.0
##  [88] whisker_0.3-2          R.oo_1.22.0            xml2_1.2.0
##  [91] biomaRt_2.38.0         compiler_3.5.2         tibble_2.0.1
##  [94] statmod_1.4.30         geneplotter_1.60.0     pcaPP_1.9-73
##  [97] gsl_1.9-10.3           RNeXML_2.3.0           stringi_1.3.1
## [100] GenomicFeatures_1.34.3 RSpectra_0.13-1        trimcluster_0.1-2.1
## [103] lattice_0.20-38        nloptr_1.2.1           pillar_1.3.1
## [106] ADGofTest_0.3          zinbwave_1.4.1         data.table_1.12.0
## [109] bitops_1.0-6           rtracklayer_1.42.1     R6_2.4.0
## [112] latticeExtra_0.6-28    hwriter_1.3.2          gridExtra_2.3
## [115] KernSmooth_2.23-15     codetools_0.2-16       boot_1.3-20
## [118] MASS_7.3-51.1          gtools_3.8.1           assertthat_0.2.0
## [121] rhdf5_2.26.2           pkgmaker_0.27          withr_2.1.2
## [124] locfdr_1.1-8           GenomeInfoDbData_1.2.0 diptest_0.75-7
## [127] hms_0.4.2              grid_3.5.2             class_7.3-15
## [130] minqa_1.2.4            rmarkdown_1.11         numDeriv_2016.8-1
```
