# Supplementary material for: Shank3 modulates sleep and expression of circadian transcription factors
Source: eLife. 2019 Apr 11;8:e42819. doi: 10.7554/eLife.42819 (PMC6488297; doi:10.7554/eLife.42819)
Supplement: Supplementary file 1. — Summary of wheel-running behavior measured for each week of 12:12 hr light:dark cycles for both wild type and Shank3∆C mice. A linear mixed-effects ANOVA was used to estimate the contributions of genotype and the interaction between genotype and time for period, alpha, and wheel-running activity for LD week 1 through 5. There is no significant interaction effect for genotype x time for period (p=0.41), but there is a significant effect of genotype (p=0.047, indicated by *). There is no significant interaction effect for genotype x time for alpha (p=0.43), but there is a significant effect of time (p=0.004, indicated by †). There is a significant interaction effect (indicated by vertical bar) for genotype x time for activity (p<0.0001). See Source data 1 for details. Values are means ± SEM for wild type (n = 8) and Shank3∆C (n = 8) mice. [file elife-42819-supp1.pdf]

	Wild Type		*Shank3$^{\Delta C}$*
		*Period (h)**	
LD Week 1	24.0 ± 0.0		21.3 ± 2.7
LD Week 2	24.0 ± 0.0		23.9 ± 0.1
LD Week 3	24.0 ± 0.0		24.2 ± 0.9
LD Week 4	24.0 ± 0.0		24.0 ± 0.0
LD Week 5	24.0 ± 0.1		24.1 ± 0.1
		Alpha (h)†	
LD Week 1	9.8 ± 0.6		8.2 ± 0.7
LD Week 2	10.0 ± 0.5		8.2 ± 0.7
LD Week 3	9.7 ± 0.5		8.3 ± 0.9
LD Week 4	9.1 ± 0.5		8.8 ± 0.7
LD Week 5	10.1 ± 0.6		9.0 ± 0.7
		Activity (rev/day)	
LD Week 1	39298.0 ± 1517.1		14509.7 ± 1273.4
LD Week 2	42867.1 ± 550.2		15256.3 ± 414.8
LD Week 3	40513.3 ± 819.3		20229.9 ± 1424.4
LD Week 4	37703.6 ± 325.1		26488.7 ± 1180.3
LD Week 5	40210.9 ± 549.5		28560.9 ± 1545.7
		**p <0.0001**	

Supplementary file 1. Wheel-running behavior in Shank3$^{\Delta C}$ mice across 5 weeks of continuous light:dark (LD) cycles. Summary of wheel-running behavior measured for each week of 12:12 h light:dark cycles for both wild type and Shank3$^{\Delta C}$ mice. A linear mixed-effects ANOVA was used to estimate the contributions of genotype and the interaction between genotype and time for period, alpha, and wheel-running activity for LD week 1 through 5. There is no significant interaction effect for genotype x time for period ($p = 0.41$), but there is a significant effect of genotype ($p = 0.047$, indicated by *). There is no significant interaction effect for genotype x time for alpha ($p = 0.43$), but there is a significant effect of time ($p = 0.004$, indicated by †). There is a significant interaction effect (indicated by vertical bar) for genotype x time for activity ($p < 0.0001$). See Supplementary file 1 – source data 1 for details. Values are means ± SEM for wild type (n = 8) and Shank3$^{\Delta C}$ (n = 8) mice.
