# Supplementary material for: Shank3 modulates sleep and expression of circadian transcription factors
Source: eLife. 2019 Apr 11;8:e42819. doi: 10.7554/eLife.42819 (PMC6488297; doi:10.7554/eLife.42819)
Supplement: Transparent reporting form [file elife-42819-transrepform.docx]

***eLife’s* transparent reporting form**

We encourage authors to provide detailed information *within their submission* to facilitate the interpretation and replication of experiments. Authors can upload supporting documentation to indicate the use of appropriate reporting guidelines for health-related research (see [EQUATOR Network](http://www.equator-network.org/%20)), life science research (see the [BioSharing Information Resource](https://biosharing.org/)), or the [ARRIVE guidelines](http://www.plosbiology.org/article/info:doi/10.1371/journal.pbio.1000412) for reporting work involving animal research. Where applicable, authors should refer to any relevant reporting standards documents in this form.

If you have any questions, please consult our Journal Policies and/or contact us: [editorial@elifesciences.org](mailto:editorial@elifesciences.org).

**Sample-size estimation**

- You should state whether an appropriate sample size was computed when the study was being designed
- You should state the statistical method of sample size computation and any required assumptions
- If no explicit power analysis was used, you should describe how you decided what sample (replicate) size (number) to use

Please outline where this information can be found within the submission (e.g., sections or figure legends), or explain why this information doesn’t apply to your submission:

Sample sizes were determined before the experiments were carried out on the basis of previous publications from our group that showed had the sufficient power to capture the effect of interest. Sample sizes were as follows:

- n=10 per group for sleep phenotyping experiments (20 total)

- n=5 per group for RNA-seq experiments (20 samples total)

- n=8 WT and n=9 mutants per group for circadian experiment (17 samples total).

All sample sizes can be found in the materials and methods section.

**Replicates**

- You should report how often each experiment was performed
- You should include a definition of biological versus technical replication
- The data obtained should be provided and sufficient information should be provided to indicate the number of independent biological and/or technical replicates
- If you encountered any outliers, you should describe how these were handled
- Criteria for exclusion/inclusion of data should be clearly stated
- High-throughput sequence data should be uploaded before submission, with a private link for reviewers provided (these are available from both GEO and ArrayExpress)

Please outline where this information can be found within the submission (e.g., sections or figure legends), or explain why this information doesn’t apply to your submission:

Each experiment was performed once; all replicates were independent biological replicates (individual mice). The data obtained for sleep recordings is provided as source data in the following file: Figures 2- source data 1 and Figure 3- source data 1.

The RNA-seq data was submitted to GEO and is publicly available (GSE113754). The processed gene expression and functional annotation data is available in Figure 4 source data 1 and Table 1 source data 1. The circadian activity data in LD:DD for individual replicates is provided in Figure 5 – figure supplement 1. Two Shank3 mutant animals were excluded as outliers in the LD:DD circadian activity study because they failed to run on the wheel for sufficient amount of time. The control circadian activity data in LD:LD is provided in Supplementary file 2. One Shank3 mutant animal was excluded as outlier in the LD:LD circadian activity study because it failed to run on the wheel for sufficient amount of time. All criteria for inclusion/exclusion in the studies are outlined in the materials and methods section.

**Statistical reporting**

- Statistical analysis methods should be described and justified
- Raw data should be presented in figures whenever informative to do so (typically when N per group is less than 10)
- For each experiment, you should identify the statistical tests used, exact values of N, definitions of center, methods of multiple test correction, and dispersion and precision measures (e.g., mean, median, SD, SEM, confidence intervals; and, for the major substantive results, a measure of effect size (e.g., Pearson's r, Cohen's d)
- Report exact p-values wherever possible alongside the summary statistics and 95% confidence intervals. These should be reported for all key questions and not only when the p-value is less than 0.05.

Please outline where this information can be found within the submission (e.g., sections or figure legends), or explain why this information doesn’t apply to your submission:

Sleep data was analyzed using SPSS as detailed in the materials and methods section. Details of the statistics performed on sleep data are available in Figure 2- source data 2 and Figure 3- source data 2. RNA-seq data and circadian data were analyzed with R. All R code used in statistical analysis in this article is available in Source Code file 1 (as a tutorial) and at <https://github.com/drighelli/peixoto>. Details of the statistics performed on circadian data are available in Table 2- source data 1 and Supplementary file 2- source data 1

(For large datasets, or papers with a very large number of statistical tests, you may upload a single table file with tests, Ns, etc., with reference to sections in the manuscript.)

**Group allocation**

- Indicate how samples were allocated into experimental groups (in the case of clinical studies, please specify allocation to treatment method); if randomization was used, please also state if restricted randomization was applied
- Indicate if masking was used during group allocation, data collection and/or data analysis

Please outline where this information can be found within the submission (e.g., sections or figure legends), or explain why this information doesn’t apply to your submission:

Animals of each genotype (WT or Shank3 mutant) were randomly allocated to each experimental group.

**Additional data files (“source data”)**

- We encourage you to upload relevant additional data files, such as numerical data that are represented as a graph in a figure, or as a summary table
- Where provided, these should be in the most useful format, and they can be uploaded as “Source data” files linked to a main figure or table
- Include model definition files including the full list of parameters used
- Include code used for data analysis (e.g., R, MatLab)
- Avoid stating that data files are “available upon request”

Please indicate the figures or tables for which source data files have been provided:

Source data can be found as follows.

A summary of the literature used as reference for Figure 1 is available in Figure 1-source data 1. The data obtained for sleep recordings is provided as Figures 2-source data 1 and Figure 3-source data 1. The RNA-seq data was submitted to GEO and is publicly available (GSE113754). Details of differentially expressed genes and functional annotation analysis of RNA-seq data can be found in Figure 4 – source data 1 and Table 1 – source data 1. The circadian activity data in LD:DD for individual replicates is provided in Figure 5 – figure supplement 1. R code can be found in Source Code file 1 and available through https://github.com/drighelli/peixoto.
